# Improved Detection of Extended Spectrum Beta-Lactamase (ESBL)-Producing *Escherichia coli* in Input and Output Samples of German Biogas Plants by a Selective Pre-Enrichment Procedure

**DOI:** 10.1371/journal.pone.0119791

**Published:** 2015-03-23

**Authors:** Thorsten Schauss, Stefanie P. Glaeser, Alexandra Gütschow, Wolfgang Dott, Peter Kämpfer

**Affiliations:** 1 Institut für Angewandte Mikrobiologie, Justus-Liebig-Universität Giessen, IFZ-Heinrich-Buff-Ring 26–32, D-35390, Giessen, Germany; 2 Institut für Hygiene und Umweltmedizin, Rheinisch-Westfälische Technische Hochschule, D-52057, Aachen, Germany; Charité-University Medicine Berlin, GERMANY

## Abstract

The presence of extended-spectrum beta-lactamase (ESBL)-producing *Escherichia coli* was investigated in input (manure from livestock husbandry) and output samples of six German biogas plants in 2012 (one sampling per biogas plant) and two German biogas plants investigated in an annual cycle four times in 2013/2014. ESBL-producing *Escherichia coli* were cultured by direct plating on CHROMagar ESBL from input samples in the range of 10^0^ to 10^4^ colony forming units (CFU) per g dry weight but not from output sample. This initially indicated a complete elimination of ESBL-producing *E*. *coli* by the biogas plant process. Detected non target bacteria were assigned to the genera *Acinetobacter*, *Pseudomonas*, *Bordetella*, *Achromobacter*, *Castellaniella*, and *Ochrobactrum*. A selective pre-enrichment procedure increased the detection efficiency of ESBL-producing *E*. *coli* in input samples and enabled the detection in five of eight analyzed output samples. In total 119 ESBL-producing *E*. *coli* were isolated from input and 46 from output samples. Most of the *E*. *coli* isolates carried CTX-M-type and/or TEM-type beta lactamases (94%), few SHV-type beta lactamase (6%). Sixty-four *bla*_CTX-M_ genes were characterized more detailed and assigned mainly to CTX-M-groups 1 (85%) and 9 (13%), and one to group 2. Phylogenetic grouping of 80 *E*. *coli* isolates showed that most were assigned to group A (71%) and B1 (27%), only one to group D (2%). Genomic fingerprinting and multilocus sequence typing (MLST) showed a high clonal diversity with 41 BOX-types and 19 ST-types. The two most common ST-types were ST410 and ST1210. Antimicrobial susceptibility testing of 46 selected ESBL-producing *E*. *coli* revealed that several isolates were additionally resistant to other veterinary relevant antibiotics and some grew on CHROMagar STEC but shiga-like toxine (SLT) genes were not detected. Resistance to carbapenems was not detected. In summary the study showed for the first time the presence of ESBL-producing *E*. *coli* in output samples of German biogas plants.

## Introduction

Intensive application of antibiotics in livestock husbandry increases the abundance of extended-spectrum beta-lactamase (ESBL)-producing *Enterobacteriaceae* in animals and in consequence in their manure [1]. The presence of ESBL-producing *E*. *coli* in manure from livestock husbandry was frequently reported [2,3]. Recent studies showed that ESBL-producing *E*. *coli* strains isolated from humans and livestock husbandry represented the same sequence types (ST types) and/ or harbored identical ESBL gene carrying plasmids. This indicates that ESBL-producing strains or ESBL genes/plasmids from livestock husbandry are potentially transmitted to humans or to human pathogenic *E*. *coli* [4]. The application of manure as organic fertilizer on fields can lead to release and widespread of antibiotic substances and antibiotic resistant bacteria into the environment [5,1]. Hartmann and colleagues [2] detected ESBL-producing *E*. *coli* that were genotypically identical (identical rep-PCR pattern) in fecal samples and the environment of farms including cultivated and pasture soils as well as composted manure. Alternatively to the direct application of manure to fields, manure and slurry in combination with energy crops are used as input materials for biogas plants to generate biofuels. The process of methane formation in biogas plants is divided into four major steps, hydrolysis, acidogenesis, acetogenesis and methanogenesis [6]. Each step is carried out by different consortia of microorganisms, which are partly interrelated syntrophically and show different environmental requirements [7]. Organic material is degraded to smaller compounds as amino acids, sugars and fatty acids by secretion of extracellular enzymes. Most of these bacteria are strict anaerobes such as *Bacterioides* and *Clostridium* species. Some facultative anaerobic bacteria as *Streptococci* and *Enterobacteriaceae* are also involved [6]. Higher volatile fatty acids are transformed into acetate and hydrogen by a group of not well characterized hydrogen-producing acetogenic microorganisms [6]. Since accumulation of hydrogen inhibits acetogenic bacteria, it has to be metabolized by methanogens to keep hydrogen partial pressure low to enable acetogenesis. Two strict anaerobe groups of methanogenic bacteria produce methane from acetate or hydrogen and carbon dioxide [6]. Biogas production is either a mesophilic (37–42°C) or thermophilic (at least 50°C) process. Anaerobic conditions and high process temperatures are given as main arguments that pathogenic bacteria are eliminated during the biogas plant process. As reported by Phillipp and Hölzle [8] *E*. *coli* were not detected in output samples of thermophilic biogas plants. Recently, the detection of *E*. *coli* after the anaerobic digestion of cattle manure was reported [9]. None of the detected isolates was resistant to a fourth-generation cephalosporin; but the isolates were not isolated from manure in the presence of cephalosporins. This non-selective isolation of resistant bacteria may result in a loss of sensitivity and underestimation of resistant bacteria. Detailed studies about the detection of ESBL-producing *E*. *coli* in output samples of biogas plants in general were not published so far. The major aim of our study was to investigate if ESBL-producing *E*. *coli* can persist the biogas plant process to assess the risk for the release of ESBL-producing *E*. *coli* with biogas plant output material into the environment. Therefore, we compared the abundance of ESBL-producing *E*. *coli* in input and output material of six mesophilic and one thermophilic German biogas plants compared to the occurrence in respective input material (liquid and solid manure mixtures from livestock husbandry). We furthermore aimed to establish an efficient cultivation-dependent detection method of ESBL-producing *E*. *coli* and compared the efficiency of a direct plating on CHROMagar ESBL with the established selective pre-enrichment method. Isolated ESBL-producing *E*. *coli* were characterized in detail by genomic fingerprinting, phylogenetic assignment, multilocus sequence typing (MLST), ESBL gene characterization and antimicrobial susceptibility testing.

## Results

### Bacterial growth on CHROMagar ESBL after direct plating

In summary ten samples, five input and five output samples, were investigated in 2012 from all five biogas plants, four mesophilic (BGA 001, 002, 005, and 006) and one thermophilic biogas plant (BGA 012) by direct plating on CHROMagar ESBL to enumerate ESBL-producing *E*. *coli*. The input and output samples of the individual biogas plants were always sampled at the same day. The concentration of bacteria cultured on CHROMagar ESBL was in the range of 10^3^ to 10^6^ CFUs (g dry weight)^-1^ for biogas plant input and 10^1^ to 10^3^ CFUs(g dry weight)^-1^ for biogas plant output samples. The concentration in output samples was always two to four orders of magnitude lower than in respective input samples, except for the thermophilic biogas plant (BGA 012). There, the concentration of culturable bacteria in the output sample was in the same range as in the input samples with 10^3^ CFUs(g dry weight)^-1^. ESBL-producing *E*. *coli* (represented by pink colonies as given in the leaflet of the CHROMagar ESBL and confirmed by partial 16S rRNA gene sequencing in our study) were determined in four input samples of the five 2012 investigated biogas plants with CFUs per g dry weight in the range of 10^1^ (BGA 002 and 005) to 10^3^ (BGA 001 and 006). ESBL-producing *E*. *coli* could not be detected in output samples. Blue colonies which represented potentially ESBL-producing *Klebsiella*, *Enterobacter* or *Citrobacter* were only cultured from an input sample of one biogas plant with 10^1^ CFUs (g dry weight)^-1^ (BGA 002). Most of the colonies were beige to brown colored and overgrew ESBL-producing *E*. *coli*. Beige to brown colonies were determined in all 2012 analyzed input samples in the range of 10^3^ to 10^6^ CFUs (g dry weight)^-1^. The concentration was 3 to 4 orders of magnitude lower in respective output samples, except for the thermophilic biogas plant BGA 012; there the concentration in input and output samples were in the same range with 10^3^ CFUs(g dry weight)^-1^.

Two of the mesophilic biogas plants (BGA 001 and 015) were subsequently investigated in a second sampling period, 2013/2014. Eight samples, four input and four output samples were investigated for each of the biogas plants. Input samples were taken for both biogas plants in February, April, July/August and October 2013 and respective output samples approximately ten weeks after input samples in April, July/August and October 2013 and February 2014 taking the transfer time through biogas plants into account. The concentration of bacteria cultured on CHROMagar ESBL was for input samples in the range of 10^4^ to 10^5^ CFUs (g dry weight)^-1^ (BGA 001) and 10^3^ to 10^6^ CFUs (g dry weight)^-1^ (BGA 015). In output samples growth was only obtained in the April sample of BGA 001 with 10^2^ CFUs (g dry weight)^-1^ and in the August and October samples of BGA 015 with 10^3^ CFUs (g dry weight)^-1^. ESBL-producing *E*. *coli* were again only detected in input samples with 10^2^ to 10^4^ CFUs (g dry weight)^-1^ (BGA 001) and 10^1^ to 10^2^ CFUs (g dry weight)^-1^ (BGA 015) with lowest concentrations in July. Blue colonies were also detected in all input samples with 10^1^ to 10^4^ CFUs (g dry weight)^-1^ but only one colony in one output sample (October, BGA 15). The major fraction of bacteria cultured on CHROMagar ESBL again grew as beige to brown colored colonies with 10^3^ to 10^6^ CFUs (g dry weight)^-1^ overgrowing ESBL-producing *E*. *coli* (S1 Fig.).

### Species identification and ESBL gene screening after direct plating

**Fig 1:**
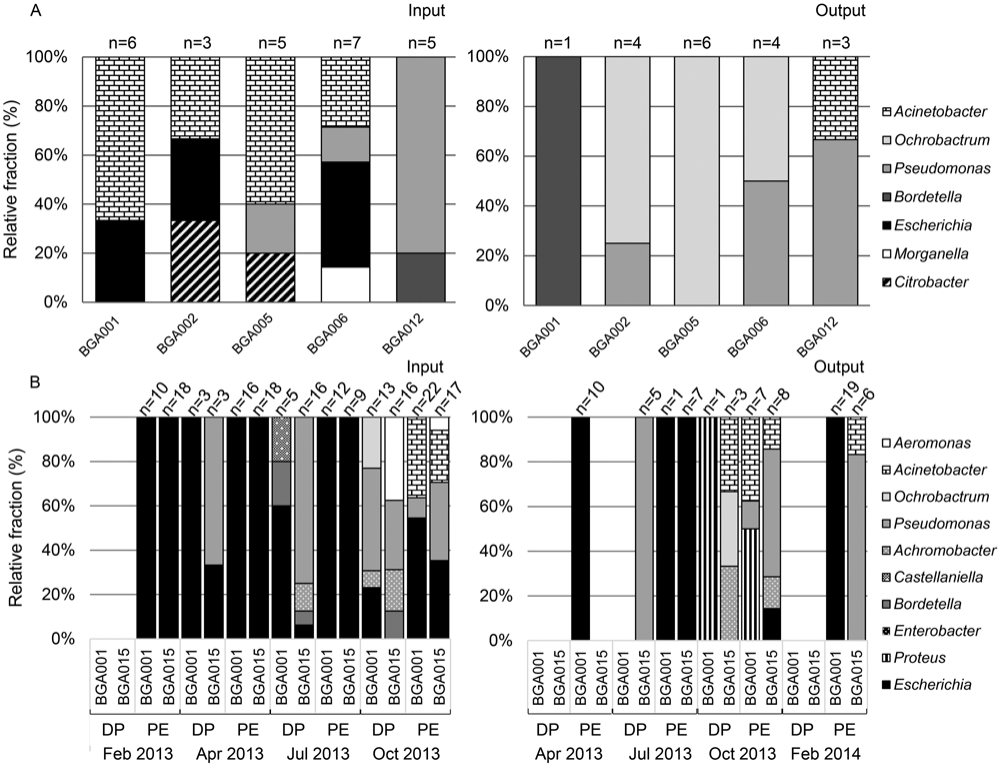
Phylogenetic identification of isolates representing most abundant colonies grown on CHROMagar ESBL after direct plating of input or output samples from German biogas plants. **A**, Five German biogas plants investigated in 2012; **B**, Two biogas plants sampled in February, July and October 2013 and February 2014. For input samples 2012 26 isolates of input and 18 isolates of output samples were identified; 2013/14, 178 isolates of input (DP: 56; PE: 122) and 72 isolates of output samples (DP: 9; PE: 63). Identification based on partial 16S rRNA gene sequence analysis (approximately 1000 nt) with assignment at the genus level based on at least 98.7% 16S rRNA gene sequence similarities to type strains of respective genera determined by BLAST analysis using the EzTaxon database. DP: direct plating on CHROMAgar ESBL; PE: selective pre-enrichment and subsequent plating on CHROMAgar ESBL. Numbers (n =) above the bars represent the number of isolates investigated per sample. The isolates were representative for abundant colonies grown on CHROMagar ESBL plates.

Bacteria representing most abundant colonies after direct plating on CHROMagar ESBL were isolated and phylogenetically identified by partial 16S rRNA gene sequencing. Sequence similarities were >98.5% to closest related type strains of described species indicating a reliable assignment at least at the genus level. As already mentioned all pink-colonies but none of the other colored colonies represented *E*. *coli* which all contained an ESBL gene, either a CTX-M-type or TEM-type gene. In 2012, a total of 44 isolates (26 from input and 18 from output samples) were identified. *E*. *coli* (6 isolates) were isolated from three out of five biogas plant input samples (Fig. 1A). Only few other *Enterobacteriaceae* were detected, one abundant blue colony morphotype was assigned to the genus *Citrobacter* (BGA 002, one isolate) and one abundant beige colony morphotype to the genus *Morganella* (BGA 006; 4 isolates; Fig. 1A). In one of the *Morganella* isolates a *bla*_CTX-M_ gene was detected. All other beige to brown colored colonies were assigned to the genera *Acinetobacter* (*Moraxellaceae*; 10 isolates), *Pseudomonas (Pseudomonadaceae*; 6 isolates) and *Bordetella* (*Alcaligenaceae*; one isolate) (Fig. 1A). *Acinetobacter* and *Pseudomonas* were the most frequently determined non-target genera identified from four out of five biogas plant input samples.

All identified colonies from output samples (all beige to brown colored) were assigned to the genera *Ochrobactrum* (*Brucellaceae*; 11 isolates), *Pseudomonas* (5 isolates), *Acinetobacter* and *Bordetella* (both one isolate). Of those, the genera *Ochrobactrum* and *Acinetobacter* were most frequently detected in four out of five biogas plant output samples (Fig. 1A). *Ochrobactrum* spp. were detected with increased abundance in output samples. The high concentration of brown colored colonies obtained in the output sample of the thermophilic biogas plant BGA 012 was due to the growth of *Acinetobacter* spp. and *Pseudomonas spp*. (Fig. 1A).

For the two annually investigated biogas plants BGA 001 and 015, 65 isolates representing most abundant colonies were identified by direct plating, 56 from input and 9 from output samples. Few *E*. *coli* (11 isolates) were cultured from seven out of eight input samples, but not from output samples. Only one isolate from an output sample was identified as a member of the *Enterobacteriaceae* (brown colonies) assigned to the genus *Proteus* (BGA 001 October). In contrast to the 2012 sampling abundant blue colored colonies represented *Aeromonas* spp. (*Aeromonadaceae*; six isolates from input and one isolate of an output sample, respectively). Abundant beige to brown coloured colonies were only phylogenetically identified from July and October samples and were assigned for input samples to the genera *Pseudomonas* and *Ochrobactrum* (23 and five isolates), as well as *Achromobacter*, *Bordetella* and *Castellaniella* (all three *Alcaligenaceae*; four, one, and seven isolates), and for output samples to the genera *Pseudomonas*, *Ochrobactrum*, and *Acinetobacter* (five, one, and one isolates; Fig. 1B). According to the text above, six *E*. *coli* isolates and one *Morganella* isolate contained CTX-M-type or TEM-type gene (sampling 2012). Further sampling (2013) revealed 11 *E*. *coli* isolates from input containing CTX-M-type + TEM-type gene (8 isolates) or a CTX-M-type gene (3 isolates). ESBL genes (*bla*_CTX-M_, *bla*_TEM_ and *bla*_SHV_) were not detected in any of the other isolates obtained by the direct plating approach.

### ESBL pre-enrichment increased the detection efficiency of ESBL-producing *E*. *coli*

**Fig 2:**
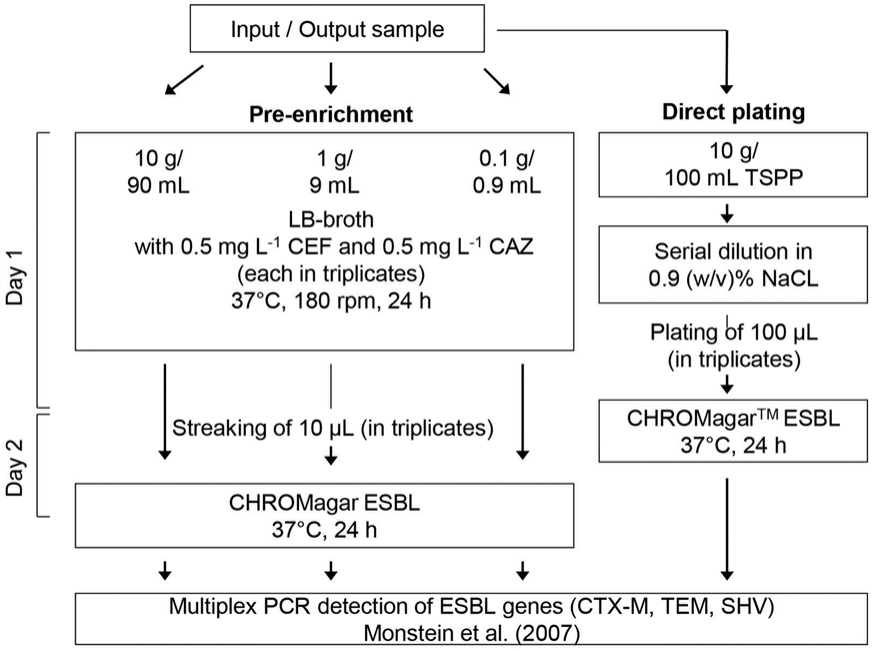
Flowsheet of the cultivation-dependent detection of ESBL-producing *Enterobacteriaceae* in input and output samples of biogas plants including a specifically established pre-enrichment method compared to direct plating (DP) on CHROMagar ESBL. For direct plating (DP), bacteria were detached from 10 g fresh samples by shaking in sterile 0.02% tetrasodium-pyrophophate buffer (TSPP) and horizontal shaken at room temperature for 5 min. After 30 min sedimentation the supernatant (10^0^) was serially diluted (up to 10^-3^) and 100 μL of each dilution step were plated in triplicates on CHROMagar ESBL. For pre-enrichment (PE), 0.1, 1, or 10 g fresh samples were pre-incubated (in triplicates) in LB broth containing 0.5 mg L^-1^ cefotaxim (CEF) and 0.5 mg L^-1^ ceftazidim (CAZ) for 24 h at 37°C. Thereafter 10 μL of the pre-enrichments were streaked on CHROMagar ESBL. After 24 h incubation at 37°C for both methods, cream, pink and blue coloured colonies were screened from the presence of CTX-M, TEM and SHV-type ESBL genes using a multiplex PCR.

Based on the low detection efficiency of ESBL-producing *E*. *coli* by direct plating on CHROMagar ESBL a selective pre-enrichment procedure was established (Fig. 2).

The pre-enrichment procedure was tested first with 10 g input and output samples parallel to direct plating on CHROMagar ESBL for BGA 006 and 012 analyzed in 2012. Plating on CHROMagar ESBL after pre-enrichment clearly showed an increased abundance of ESBL-producing *E*. *coli* on CHROMagar ESBL plates for input samples and enabled the detection in output samples of BGA 006 in contrast to the direct plating approach. Two ESBL-producing *E*. *coli* were obtained from the input and one from the output sample. The *E*. *coli* isolates from the input sample contained a CTX-M-type or a CTX-M-type + TEM-type gene and the *E*. *coli* isolates from the output samples a CTX-M-type + TEM-type gene. Blue colored colonies from input samples represented *Enterobacter* sp. closest related to *Enterobacter aerogenes* carrying a *bla*_CTX-M_ gene. For the thermophilic biogas plant BGA 012 ESBL-producing *E*. *coli* were not detected, neither in input nor in output sample, which was in line with the direct plating approach. In none of the beige to brown colored colonies an ESBL gene could be detected.

In the second sampling period, 2013–2014, for each sample (4 input and 4 output samples of both biogas plants) pre-enrichments of 0.1, 1 and 10 g fresh material were performed (each in triplicates, Fig. 2) in parallel to the direct plating approach. Even the visual comparison of growth on CHROMagar ESBL plates after direct plating and pre-enrichment illustrated the increased detection efficiency of ESBL-producing *E*. *coli* from input samples of the investigated biogas plants (S1 Fig.). ESBL-producing *E*. *coli* were detected in all eight analyzed input samples after pre-incubation of 10 to even 0.1 g fresh input material. From the February 2013 input sample of BGA 001 ESBL-producing *E*. *coli* were only detected after pre-enrichment of 1 and 10 g samples indicating a lower abundance of ESBL-producing *E*. *coli* compared to the other input samples. At all 101 ESBL-producing *E*. *coli* isolates were obtained in input samples, 50 from BGA 001 and 51 from BGA 015. ESBL-producing *E*. *coli* from BGA 001 either contained a CTX-M-type gene (27 isolates), a CTX-M + TEM-type gene (22 isolates) and one only a TEM-type gene. Most of the ESBL-producing *E*. *coli* from BGA 015 contained also a CTX-M-type gene (39 isolates) or a CTX-M-type + TEM-type gene (7 isolates), but few also contained a SHV-type gene (3 isolates), or a TEM-type + SHV-type gene (2 isolates).

The application of the pre-enrichment method enabled also the detection of ESBL-producing *E*. *coli* in five out of the eight analyzed output samples; in two output samples only after pre-enrichment of 10 g (July BGA 001 and October BGA 015), in one output sample after pre-enrichment of 1 and 10 g (April BGA 001) and in two output samples even after pre-enrichment of 0.1 g (February 2014 BGA 001; July BGA 015). A total of 43 ESBL-producing *E*. *coli* were isolated from output samples, 34 from BGA 001 and 8 from BGA 015. In contrast, ESBL-producing *E*. *coli* were not cultured after direct plating on CHROMagar ESBL. The *E*. *coli* isolates either contained a CTX-M-type gene (24 isolates from BGA 001; 7 from BGA 015) or a CTX-M-type + TEM-type gene (10 isolates from BGA 001; 1 isolate from BGA 015).

Beside the ESBL-producing *E*. *coli* few blue colored colonies were also detected, which were again identified as *Aeromonas* spp. Beige to brown coloured colonies that grew beside ESBL-producing *E*. *coli* on the CHROMAgar medium were again assigned to the genera *Acinetobacter*, *Pseudomonas* and *Achromobacter*. *Acinetobacter* spp. and *Pseudomonas* spp. were detected in input and output samples also after pre-enrichment; *Achromobacter* spp. only in output samples of both biogas plants (Fig. 1B). *Aeromonas* spp. were detected in input and *Proteus* spp. in output samples of BGA 001 and BGA 015, respectively. *Bla*_CTX-M_-, *bla*_TEM_- and *bla*_SHV_ genes were not detected in the *Proteus* spp. Compared to the direct plating on CHROMagar ESBL the fraction of ESBL-producing *E*. *coli* isolates was increased significantly in all samples (input and output; BGA 001 and BGA 015) (Fig. 1B). In contrast to the direct plating approach *Acinetobacter* spp. were detected in all samples after pre-enrichment and *Pseudomonas* spp. in the same samples after direct plating. In contrast, *Bordetella* and *Ochrobactrum* spp. were not detected and *Aeromonas* spp. only in few of the samples after specific pre-enrichment (Fig. 1B).

### Strategies for detailed characterization of ESBL-*E*. *coli* isolates

Taking both sampling periods together, 163 *E*. *coli* isolates carrying ESBL genes (120 from input and 43 from output samples) were isolated from CHROMagar ESBL. Seventeen of the isolates were detected after direct plating on CHROMagar ESBL (all from input samples) and 146 after the application of the selective pre-enrichment, of those 103 isolates originated from input and 43 isolates from output samples. All of those isolates were screened by BOX-PCR. If identical BOX-pattern were obtained for isolates originating from the same sample, only one of isolates was further investigated to avoid working with clones of the same *E*. *coli* strain. Based on this selection 80 *E*. *coli* isolates were further typed with phylogenetic *E*. *coli* grouping. Further, at least one representative of each BOX-genotype (considering isolates from input and output samples of different biogas plants) was selected for MLST analysis and antimicrobial susceptibility testing.

### Distribution of *E*. *coli* genotypes in input and output samples of biogas plants

The comparison of the 163 ESBL-producing *E*. *coli* isolates by genomic fingerprinting (BOX-PCR) resulted in 41 different BOX-genotypes. Among those several were highly similar, and showed only slight differences. BOX-genotypes were used to investigate the distribution pattern of *E*. *coli* strains among input and output samples investigated between 2012 to 2014 (S2 Fig.). In general 1 to 7 different BOX-genotypes were obtained within one input and 1 to 8 different genotypes within one output sample. Twenty-six of the detected BOX-genotypes occurred only in one of the samples (63% of the BOX-genotypes; S2 Fig.). Among the 41 BOX-genotypes eight were detected in input and output samples and eight only in output samples. In 2012 only six BOX-genotypes were detected among those one which was obtained again in two different input samples in 2013 (BOX-genotype 2b). With the application of the pre-enrichment procedures in 2013 an increased diversity of BOX-genotypes were obtained. At all eight different BOX-genotypes were detected in both, input and output samples; nine BOX-genotype only in an output sample. The other BOX-genotypes occurred unequally distributed among the samples. Only one BOX-genotype was detected in an input sample and eight weeks later in the respective output sample (BOX-genotype 3a; S2 Fig.) indicated a transfer through the biogas plant. However, the isolates with the same BOX-genotype carried different *bla*_CTX-M_ genes, the input isolates CTX-M-group 1 genes; the output isolate a CTX-M-group 9 gene, which might indicate a gene transfer/exchange in the biogas plant process.

### Sequence based characterization of *bla*_CTX-M_, *bla*_TEM_ and *bla*_SHV_ genes

PCR screening of 163 ESBL-producing *E*. *coli* revealed presence of *bla*_CTX-M_ genes (66%, 101 isolates) or a *bla*_CTX-M_ + *bla*_TEM_ gene (31%, 47 isolates); only *bla*_TEM_ gene (1 isolate), only *bla*_SHV_ gene (3 isolates), and *bla*_TEM_ + *bla*_SHV_ genes (2 isolates). From 71 of 163 ESBL-producing *E*. *coli* isolates ESBL genes were further characterized by sequence analysis. Among those, 67 *E*. *coli* contained a *bla*_CTX-M_ and partially additionally a *bla*_TEM_ gene, one with a *bla*_TEM_ gene only, one with a *bla*_SHV_ gene, and two with a *bla*_SHV_ and *bla*_TEM_ gene. In summary 67 *bla*_CTX-M_, 12 *bla*_TEM_ and 3 *bla*_SHV_ genes were characterized by sequence analysis. Analysis based on respective amino acid sequences were used to assign the detected *bla*_CTX-M_ genes to main CTX-M-gene groups, most were assigned to the CTX-M-1 group (85.1%; 57 of 67 genes), followed by the CTX-M-9 group (13.4%; 9 of 67 genes) and with very low abundance the CTX-M-2 group (1.5%; 1 of 67 gene) (S3 Fig.). Within the CTX-M-1 group genes were further differentiated by comparing indicator amino acids. The analysis of partial sequences indicated the presence of *bla*_CTX-M-1_, *bla*_CTX-M-15_, and *bla*_CTX-M-32_ based on distinct clustering. Three of the analyzed *bla*_CTX-M_ genes were identical to *bla*_CTX-M-1_ (5%), 12 to *bla*_CTX-M-15_ (20.3%), and 10 to *bla*_CTX-M-32_ (17%), if differences at amino acid positions aa 286, 289 were not considered. Eleven of the genes assigned to *bla*_CTX-M-15_, showed an exchange of aa268, 289 (ten: isoleucine, valine-> two serine; one: isoleucine, valine-> serine, tyrosine). In addition, all of the genes assigned to *bla*_CTX-M32_ also showed the respective amino acid exchanges (aa 286, 287: isoleucine, valine-> two serine). The rest of the CTX-M-1 group genes (15 genes, 22.4%) could not be exactly assigned to any of the three types. Closest related gene types are depicted in the amino acid based phylogenetic tree shown in S3 Fig. The six genes assigned to the CTX-M-9 group were compared more detailed with respect to amino acid 234 to differentiate between *bla*_CTX-M-9_ (aa 234: alanine) and *bla*_CTX-M-14_ (aa 234: valine) all of the tested genes contained the aa present in *bla*_CTX-M-9_.

*Bla*_CTX-M_ genes of the *Morganella* sp. and the two *Enterobacter* spp. isolates were identified as CTX-M-1 group genes, whereas *bla*_CTX-M_ gene of the four *E*. *coli* obtained from the same sample (input sample of BGA 006) carried a CTX-M-9 group gene. Three of the four *E*. *coli* showed different BOX-pattern. Based on those data, an indication of ESBL gene transfer between *E*. *coli* and other *Enterobacteriaceae* could not be obtained, but between *Morganella* spp. and *Enterobacter* spp. isolates an exchange cannot be excluded.

A selection of TEM-type genes (12 genes) were characterized based on nearly full-length amino acid sequences (position 21 to 282). Most of them (10 genes) did not show any differences to the amino acid sequence of the non-ESBL *bla*_TEM-1_. Only two TEM-type genes were clearly identified as ESBL genes. One TEM-type gene was assigned based on partial sequence to ESBL gene *bla*_TEM-159_. The *E*. *coli* which carried this ESBL *bla*_TEM_ gene had in addition an ESBL gene of the CTX-M-1 group. The TEM gene which was detected in an *E*. *coli* which carried only a TEM gene was identified as ESBL *bla*_TEM-52_. All three detected *bla*_SHV_ genes were identified as ESBL gene *bla*_SHV-12_.

### Phylogenetic *E*. *coli* grouping

Based on BOX-type selection, 80 ESBL producing *E*. *coli* isolates were assigned to phylogenetic *E*. *coli* groups A, B1, B2 and D according to Clermont et al. [10]. Most of the isolates were assigned to group A (57 isolates; 65% input; 85% output), followed by group B1 (22 isolates; 33% input; 15% output) and only one isolate of an input sample to group D. None of the isolate was assigned to group B2.

### MLST analysis of selected ESBL-producing *E*. *coli* isolates

**Fig 3:**
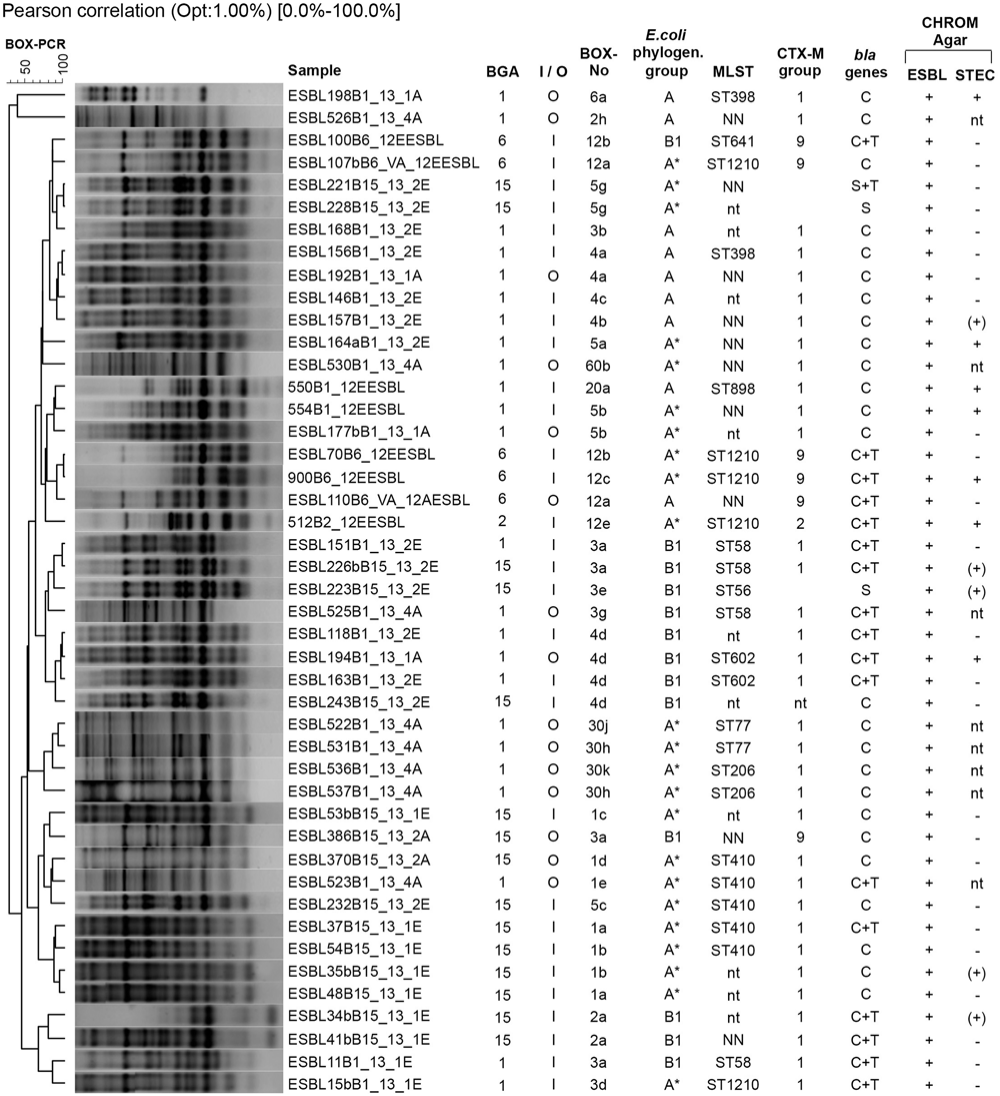
Differentiation of ESBL-producing *E*. *coli* isolates by molecular fingerprinting using BOX-PCR and detailed characterization of respective isolates. Cluster analysis was performed in Gel Compare II (Applied Maths) with UPGMA clustering based on a similarity matric calculated using the Pearson correlation (1.0% optimization and 1.0% position tolerance). Characterization of the isolates included the determination of ST-types (MLST analysis), phylogenetic *E*. *coli* typing, ESBL-gene characterization. Growth on CHROMagar ESBL and STEC. CTX-M groups were assigned by sequencing CTX-M-type genes. *E*. *coli* phylogenetic groups were determined by multiplex PCR and a dichotomous decision tree. *: assigned in group A was but there was a *yjaA* gene present. C: CTX-M; T: TEM; +: positive;-: negative. BGA shows to which biogas plant isolates belong; I = input sample, O = output sample. Names of the strains include information of the origin of the isolates: pre-enrichment (ESBL before strain number) Bxx (Biogas plant shortcut)_12, 13 or 14 (year of cultivation/isolation: either 2012, 2013 or 2014)_input or output.

A total of 35 isolates (representing 26 different BOX-genotypes) were investigated by MLST analysis. The isolates represented 19 different ST types (Table 1; Fig. 3, S4 Fig.) including nine already described ST-types, ST56 (one isolate), ST58 (four isolates), ST77 (two isolates), ST398 (two isolates), ST410 (five isolates), ST 206 and ST602 (each two isolates), ST641 and ST898 (each one isolate), and ST1210 (five isolates) and seven new ST-types. Among the nine described ST-types six were assigned to ST complexes, ST56 and ST58 to ST155 Cplx, ST398 to ST398 Cplx, ST410 to ST23 Cplx, ST77 and ST602 to ST446 Cplx and ST641 to ST86 Cplx. Four of the ST types, ST58, ST410, ST398, and ST602, were determined in both, input and output samples, four further undefined ST-types were additionally determined in output samples (Table 1; Fig. 3; S4 Fig.). Isolates that belonged to the same ST types partially represented different but highly similar BOX-genotypes but were in general assigned to the same phylogenetic *E*. *coli* groups and contained partially different sets of ESBL genes (*bla*_CTX-M_, *bla*_TEM_, *bla*_CTX-M_ and *bla*_TEM_ or *bla*_SHV_) and among the *bla*_CTX-M_ genes, genes of different CTX-M-groups, group 1, 2, or 9 (Table 1). Comparative analysis of MLST data, phylo-grouping and ESBL gene identification showed for example that isolates identified as ST1210 that originate from input samples of different biogas plants (BGA 001 and 006) carried ESBL genes of different CTX-M types. A potential transfer of isolates of the same ST-type/*E*. *coli* phylogroup with an ESBL gene of the same CTX-M group was indicated twice, for three isolates of ST410/A* (A, but with *yiaA* detection) and three isolates of ST58/B1 all carrying CTX-M-1 group genes. ST410 isolates were isolated from two input and a subsequent output sample of BGA 001, and the isolates of ST58 of two input and a subsequent output samples of BGA 015. Isolates with an identical assignment however were also detected from other BGAs indicating a more common abundance of those in manure or biogas plant output samples. An example of a stable presence of a specific ST type in a specific livestock husbandry or even a biogas plant reactor over a longer time period was indicated by the two isolates identified as ST398/A carrying an ESBL gene of the CTX-M-1 group, because the isolates were isolated in 2012 from an input samples of BGA 001 and in 2013 from an output sample of the same biogas plant.

**Table 1:** Overview of the detected *E*. *coli* ST types and the affiliation to ST Complexes determined by MLST based on seven genes, *adk*, *fumC*, *purA*, *recA*, *gyrB*, *icd*, and *mdh* using the *E*. *coli* database (http://mlst.warwick.ac.uk/mlst/dbs/Ecoli/).

	BGA	Input / Output	Time of sampling	*E*.*coli*. phylo group	Box type	*bla* gene	CTX-M group	ST	ST Complex	*adk*	*fumC*	*gyrB*	*icd*	*mdh*	*purA*	*recA*
ESBL223B15_13_2E	015	I	Apr 2013	B1	3e	S	-	ST56	ST155 Cplx	6	4	4	18	24	5	14
ESBL11B1_13_1E	001	I	Feb 2013	B1	3a	C+T	1	ST58	ST155 Cplx	6	4	4	16	24	8	14
ESBL151B1_13_2E	001	I	Apr 2013	B1	3a	C+T	1	ST58	ST155 Cplx	6	4	4	16	24	8	14
ESBL226bB15_13_2E	015	I	Apr 2013	B1	3a	C+T	1	ST58	ST155 Cplx	6	4	4	16	24	8	14
ESBL156B1_13_2E	001	I	Apr 2013	A	4a	C	1	ST398	ST398 Cplx	64	7	1	1	8	8	6
ESBL198B1_13_1A	001	O	Apr 2013	A	6a	C	1	ST398	ST398 Cplx	64	7	1	1	8	8	6
ESBL37B15_13_1E	015	I	Feb 2013	A*	1a	C+T	1	ST410	ST23 Cplx	6	4	12	1	20	18	7
ESBL54B15_13_1E	015	I	Feb 2013	A*	1b	C	1	ST410	ST23 Cplx	6	4	12	1	20	18	7
ESBL232B15_13_2E	015	I	Apr 2013	A*	5c	C	1	ST410	ST23 Cplx	6	4	12	1	20	18	7
ESBL370B15_13_2A	015	O	July 2013	A*	1d	C	1	ST410	ST23 Cplx	6	4	12	1	20	18	7
ESBL163B1_13_2E	001	I	Apr 2013	B1	4d	C+T	1	ST602	ST446 Cplx	6	19	33	26	11	8	6
ESBL194B1_13_1A	001	O	Apr 2013	B1	4d	C+T	1	ST602	ST446 Cplx	6	19	33	26	11	8	6
ESBL100B6_VA_12EESBL	006	I	Sept 2012	B1	12a	C+T	9	ST641	ST86 Cplx	9	6	33	131	24	8	7
550B1_12EESBL	001	I	July 2012	A	20a	C	1	ST898	None	6	8	4	159	9	23	7
512B2_12EESBL	002	I	July 2012	A*	12e	C+T	2	ST1210	None	187	11	4	8	8	8	2
900B6_12EESBL	006	I	Sept 2012	A*	12c	C+T	9	ST1210	None	187	11	4	8	8	8	2
ESBL70B6_12EESBL	006	I	Sept 2012	A*	12b	C+T	9	ST1210	None	187	11	4	8	8	8	2
ESBL107bB6_VA_12EESBL	006	I	Sept 2012	A*	12a	C	9	ST1210	None	187	11	4	8	8	8	2
ESBL15bB1_13_1E	001	I	Feb 2013	A*	3d	C+T	1	ST1210	None	187	11	4	8	8	8	2
554B1_12EESBL	001	I	July 2012	A*	5b	C	1	NN	None	n.d.	11	5	8	8	18	2
ESBL110B6_12AESBL	006	O	Sept 2012	A	12a	C+T	9	NN	None	187	27	5	10	12	9	2
ESBL41bB15_13_1E	015	I	Feb 2013	B1	2a	C+T	1	NN	None	n.d.	4	87	96	70	58	2
ESBL157B1_13_2E	001	I	Apr 2013	A	4b	C	1	NN	None	187	27	n.d.	10	12	n.d.	2
ESBL164aB1_13_2E	001	I	Apr 2013	A*	5a	C	1	NN	None	187	11	292	1	8	8	2
ESBL192B1_13_1A	001	O	Apr 2013	A	4a	C	1	NN	None	8	7	1	1	8	8	6
ESBL221B15_13_2E	015	I	Apr 2013	A*	5g	S+T	-	NN	None	187	n.d.	4	8	8	8	2
ESBL386B15_13_2A	015	O	July 2013	B1	3a	C	9	NN	None	n.d.	45	41	42	5	32	2
ESBL522B1_13_4A	001	O	Feb 2014	A*	30j	C	1	ST77	ST206 Cplx	6	7	5	1	8	18	27
ESBL523B1_13_4A	001	O	Feb 2014	A*	1e	C+T	1	ST410	ST23 Cplx	6	4	12	1	20	18	7
ESBL525B1_13_4A	001	O	Feb 2014	B1	3g	C+T	1	ST58	ST155 Cplx	6	4	4	16	24	8	14
ESBL526B1_13_4A	001	O	Feb 2014	A	2h	C	1	NN	None	6	25	4	140	24	239	2
ESBL530B1_13_4A	001	O	Feb 2014	A*	60b	C	1^+^	NN	None	84	11	4	8	7	8	2
ESBL531B1_13_4A	001	O	Feb 2014	A*	30h	C	1	ST77	ST206 Cplx	6	7	5	1	8	18	27
ESBL536B1_13_4A	001	O	Feb 2014	A*	30k	C	1	ST206	ST206 Cplx	6	7	5	1	8	18	2
ESBL537B1_13_4A	001	O	Feb 2014	A*	30h	C	1	ST206	ST206 Cplx	6	7	5	1	8	18	2

NN: ST type which was not present in the MLST database. n.d: Sequence type not present in reference database. Phylogenetic group assignment was done by multiplex PCR, A*: *E*. *coli* assigned to group A but a *yjaA* gene was also present. S: *bla*_SHV_, T: *bla*_TEM_, C: *bla*_CTX-M_ (multiplex PCR),

^+^ not sequenced; identified by group specific CTX-M primer; n.d.: non defined.

### Antimicrobial susceptibility testing of selected *E*. *coli* isolates

A total of 46 ESBL-producing *E*. *coli* isolates originating from various input and output samples (including the isolates analyzed by MLST) were investigated with respect to their resistance to 12 different mainly veterinary relevant antibiotics covering five main groups of antibiotics. All *E*. *coli* strains showed a typical ESBL phenotype with high MIC values of cephalosporins (ceftiofur, cefquinome), an inhibition of the resistance in the presence of clavulanic acid. All isolates showed a high resistance to the penicillin antibiotics amoxicillin (MIC > 64 mg L^-1^) and oxacillin (MIC > 32 mg L^-1^) and the macrolide tylosin (MIC > 16 mg L^-1^) as it is either intrinsic (oxacilin, tylosin) or induced in ESBL phenotypes (amoxicillin). Furthermore 74% of the isolates showed resistance to highest tested concentrations of sulfamethoxazol (MIC > 256 mg L^-1^), 54% to tetracycline (MIC > 32 mg L^-1^), and 48% to trimethoprim/ sulfamethoxazole (MIC > 16 mg L^-1^) whereas 28% of the isolates were susceptible to the lowest applied concentration of this antibiotics (MIC < 0.125 mg L^-1^). In addition 28% of the tested isolates were resistant to the highest concentration of enrofloxacin (MIC values > 8 mg L^-1^) and 63% were susceptible (MIC < 0.0625 mg L^-1^). Only for some of the tested antibiotic officially values for resistance were available in the EUCAST database. Based on these clinical breakpoints, 100% of the tested isolates (n = 46) were resistant to amoxicillin, 61% (n = 28) were resistant to tetracycline and 50% (n = 23) were resistant to trimethoprim/ sulfamethoxazole.

### Carbapenem resistance and Shigatoxin production of *E*. *coli* isolates

A selection of ESBL-producing *E*. *coli* isolates (37 isolates; including all MLST type analyzed isolates) were tested for growth on CHROMagar KPC and STEC. They were screened for the presence of carbapenemases (KPC-, NDM-, GES-, OXA-48-, IMP-, and VIM-type genes) and shiga-like-toxin genes SLT-I and SLT-II by multiplex-PCR analysis. All 37 tested ESBL-producing *E*. *coli* isolates did not grow on CHROMagar KPC and only induced a pink coloration of the agar at the area were isolates were inoculated. Screening for carbapenemase genes was negative. In addition all tested isolates were susceptible to meropenem (MIC values < 0.0625 mg L^-1^).

Eighteen of the 37 *E*. *coli* isolates formed pink colonies on CHROMagar STEC medium indicating the expression of shiga-like-toxin (*slt*). However, *slt* genes could not be detected by PCR amplification (Fig. 3).

## Discussion

In this study we applied a selective pre-enrichment procedure that increased the detection efficiency of ESBL-producing *E*. *coli* in input samples of biogas plants (mixed liquid and solid manure samples from livestock husbandry) and enabled the detection in respective output samples. ESBL-producing *E*. *coli* were the predominating ESBL-producing *Enterobacteriaceae* detected within this study. Beside, only two ESBL-producing *Enterobacter* spp. and one ESBL-producing *Morganella* sp. were detected. By direct plating of bacterial cells detached from sample material on CHROMagar ESBL, ESBL-producing *E*. *coli* were only detected in input samples which indicated falsely a complete elimination of ESBL-producing *E*. *coli* by a mesophilic biogas plant process. Most problematic by the direct plating approach was the overgrowth of the *E*. *coli* by non-target bacteria. The established pre-enrichment procedure did not enable the complete suppression of non-target organism, but resulted in an increased relative abundance of ESBL-producing *E*. *coli* compared to non-target bacteria. Non-target bacteria still grew after pre-enrichment were identified as *Pseudomonas* spp., *Acinetobacter* spp. and *Achromobacter* spp. Only ESBL-producing *E*. *coli* formed pink colonies on the applied CHROMagar ESBL. The medium specificity for ESBL-producing *E*. *coli* detection was thereby confirmed. In contrast, a clear identification of *Enterobacter* and *Citrobacter* (blue colored colonies) in general required the subsequent identification by e.g. phylogenetic identification (16S rRNA gene sequencing) because other non-target bacteria as *Aeromonas* spp. also appeared as blue colored colonies.

The high abundance of non-target bacteria on the cephalosporin containing CHROMagar ESBL after direct plating and also after selective pre-enrichment was not linked to the presence of *bla*_CTX-M_, *bla*_TEM_, or *bla*_SHV_ genes. Growth of non-target bacteria can be explained by several mechanisms, e.g. the presence of genome encoded AmpC beta lactamase known for *Ochrobactrum anthropi* [11,12], for *Pseudomonas aeruginosa* (e.g. [13]) or *Acinetobacter* spp. [14]. In our study, especially the detection of cephalosporin resistant *Acinetobacter* isolates was of concern because several of the isolates (also obtained from output samples) were assigned to the clinically relevant *Acinetobacter* species complex containing *A*. *baumannii* (>99.8% partial 16S rRNA gene sequence similarity). Taking the biogas plant process into account high amounts of cephalosporin resistant *Acinetobacter* spp. and *Pseudomonas* spp. were detected in output samples of BGA 012, a biogas plant with a thermophilic process management which was in contrast to other investigated biogas plants only fed by chicken manure. Our data indicated that a thermophilic biogas plant process (here applied in the investigated BGA 012) even increased the abundance of *Acinetobacter* species in output samples. Because ESBL-producing *E*. *coli* were not detected in input samples of the thermophilic biogas plant, neither by the direct plating nor by the pre-enrichment based cultivation, a statement to the elimination of ESBL-producing *E*. *coli* in thermophilic biogas plants cannot be given.

Growth of non-target bacteria by cultivation of bacteria on CHROMagar ESBL as *Pseudomonas aeruginosa* (AmpC producers) was already reported by Grohs et al. [15] and Saito et al. [16]. The here established pre-enrichment procedure enhanced the detection efficiency of ESBL-producing *E*. *coli* but still could not inhibit growth of non-target bacteria. Alternatively to CHROMagar ESBL Randall and colleagues [17] tested a more specific CHROMagar CTX-M medium for the isolation of CTX-M carrier from feces of farm animals. In contrast to the CHROMagar ESBL, AmpC producers should be inhibited in growth on that medium, e.g. *Pseudomonas aeruginosa*. Because of the specificity of the identification of ESBL-producing *E*. *coli*, we did not change the medium within this study, but enhanced the abundance of ESBL-producing *E*. *coli* before plating on the agar medium by using the selective pre-enrichment step.

Input samples of biogas plants where ESBL-producing *E*. *coli* were detected in our study included slurry from fatting pigs, (dairy) cattle, and breeding sows and manure from (dairy) cattle, laying hens, chicken and horses. Because only mixed samples were investigated the origin of the ESBL-producing *E*. *coli* could not be determined more exactly. The detection of ESBL-producing *E*. *coli* in German farms was recently also reported [1,18,19]. Friese et al. [1] detected among 50 investigated farms ESBL-producing *E*. *coli* in all fecal specimen of broiler farms and in more than 50% of the samples from breeding pigs and dairy cattle herds and more than 40% of the samples from fattening pig holdings. That study clearly indicated the presence of ESBL-producing *Enterobacteriaceae* in healthy pigs, turkeys and broilers in Germany. Furthermore, two ESBL-producing *E*. *coli* were detected in samples of fertilized field surfaces and few also in slurry samples supporting the transfer of culturable ESBL-producing *E*. *coli* into the environment [1]. Laube and colleagues detected ESBL-producing *E*. *coli* in broiler flocks with increased abundance during the fattening period of the broiler flocks [18]. Both colonized day-old chicks and the contaminated farm environment were mentioned as significant sources for the spread of ESBL-producing *E*. *coli* in Germany. In the study performed by Schmid and colleagues ESBL-producing *E*. *coli* were detected in 87.6% of investigated dairy cow and beef cattle farms in fecal samples, boot swaps and dust samples [19]. In addition Kola and colleagues detected an alarming high number of ESBL-producing *E*. *coli* in German broiler meat samples (>40%) furthermore indicating the spread of resistant bacteria from livestock husbandry into the environment and the human food chain [20]. Our study showed to our knowledge for the first time the detection of ESBL-producing *E*. *coli* in output samples of German biogas plants indicating that ESBL-producing *E*. *coli* can persist the biogas plant process. Most interestingly, not only *E*. *coli* survived as already reported recently [9] the biogas plant process but *E*. *coli* also remained the ESBL-genes and in consequence the cephalosporin resistance. Compared to other antibiotics cephalosporins are instable indicating that the selective pressure is getting lost during the biogas plant process [21], but as shown here ESBL-genes remained in *E*. *coli* also in absence of the selective pressure. Due to the location of ESBL genes on plasmids together with various other resistance genes (e.g. other antibiotics, heavy metals, disinfectants) ESBL-producing *E*. *coli* might be co-selected by various other substances during the processes in biogas plants.

In the present study, at total of 120 ESBL-producing *E*. *coli* isolates were isolated from mixed manure of German livestock husbandry and 43 from output samples of German biogas plants. Most of the *E*. *coli* isolates were assigned to phylogenetic groups A and B1 and only one to group D. Tenaillon et al. [22] reported group A *E*. *coli* as dominating phylogenetic group in humans and B1 in domestic animals, in contrast, in wild life animals group A *E*. *colis* were more dominant and B2 *E*. *coli* occurred with reduced abundance compared to domesticated animals. In a resent investigation of human and animal associated ESBL-producing *E*. *coli* isolated in Hesse group A and B1 *E*. *coli* were detected as most dominating in domesticated animals including horses, and group A, B1, B2 and to less extend D in human populations [23]. Tenaillon et al. [22] described phylogroup specific differences of third-generation cephalosporin resistance, group A and D were more tolerant of resistance development, whereas group B2 strains are less resistant. This corresponds with the results of our study, 57% of investigated *E*. *coli* strains were assigned in group A and except three isolates, all showed ceftiofur MIC values >32 mg L^-1^ and expect 6 isolates cefquinom MIC values >32 mg L^-1^. But in general, *E*. *coli* of all phylogenetic groups showed high MIC values of tested cephalosporins. Günther et al. [24] mention group B2 and D as “extraintestinal pathogenic group”, in this study only one isolate was assigned to group D. Furthermore group B2 is mainly reported for *E*. *coli* causing nosocomial urinal tract infections (UTI) [25]; this group was not detected in our study.

Most of the *E*. *coli* isolated obtained in our study carried more than one ESBL gene, mainly a *bla*_CTX-M_ and *bla*_TEM_ gene, two isolates even all three ESBL genes. The high abundance of isolates harboring both a *bla*_CTX-M_ and *bla*_TEM_ gene was also reported previously [26,27,28,29]. Detected *bla*_TEM_ genes seemed to represent non-ESBL *bla*_TEM-1_. The high abundance of those was also reported previously [23]. Among the identified *bla*_CTX-M_ genes most were assigned to the CTX-M-1 and CTX-M-9 group (96% of the detected *bla*_CTX-M_ genes). This was in line with the finding of Laube et al. [18] which also detected mainly CTX-M-1 group ESBL genes, followed by CTX-M-9 group ESBL genes in mixed dairy and beef cattle farms and beef cattle farms in Germany. CTX-M-1 group ESBL genes were also detected as a highly prevalent CTX-M group in other studies performed in Germany, in human intestine tract colonizing *E*. *coli* [30] and a case control study performed at a hospital [31] or by the investigation of human and domesticated animal associated isolates [23]. Among the CTX-M-1 group genes detected by Schmiedel et al. [23] *bla*_CTX-M-1_ and *bla*_CTX-M-15_ genes were the most abundant gene types in the human and animals. *Bla*_CTX-M-15_ was dominating among horse isolates. In our study partial ESBL gene sequence analysis indicated the detection of mainly *bla*_CTX-M-1_, *bla*_CTX-M-15_ and also *bla*_CTX-M-32_ genes. The CTX-M-1 group is in general the most prevalent ESBL group among animals in Europe [4]. The three detected *bla*_SHV_ genes were assigned to the *bla*_SHV-12_ type which was detected as most abundant *bla*_SHV_ gene type in flies, manure and rinsed water of poultry farms studied in the Netherlands [32]. In general *bla*_SHV-12_ is reported to be the third most abundant ESBL gene type detected in *E*. *coli* originating from food-producing and companion animals [4].

Several studies already described the presence of the genetically identical ESBL-producing *E*. *coli* isolates originating from livestock husbandry and humans [33,4]. The necessity to investigate the role of the transmission via the food chain from livestock husbandry and humans is clearly indicated. Genomic fingerprinting and MLST analysis indicated a high clonal diversity among the detected ESBL-producing *E*. *coli* isolated in our study. Several new but also several already known ST types were detected, but the worldwide pandemic community-associated *E*. *coli* clone ST131 [34] was not detected in our samples originating from livestock husbandry as expected. CTX-M-1 group *bla*_CTX-M_ gene carrying *E*. *coli* of ST58 were for example previously detected in France from pasture soil and composted manure [2] indicating their persistence in the environment, in patients in Chile [35], and in several zoo animals in Czech Republic [36]. *E*. *coli* strains of the ST410 (STC23) were described as a globally distributed ST type in human populations and animals in Europe [37,36]. Ewers and colleagues confirmed the occurrence of ST410 in livestock husbandry, wild and companion animals and humans of samples collected in Germany [37]. Among the ST410 *E*. *coli* pathogenic strains were determined, e.g. uropathogenic strains obtained in companion animals [38,39]. *E*. *coli* isolates of ST410 (all carring the same CTX-M-group genes) were determined in input and output samples of BGA 015 indicating a transfer through the biogas plant and also in the output sample of BGA 001. A ST410 *E*. *coli* strain was detected in a fecal specimen of a patient diseased on HUS in Germany in 1999 [40]. ESBL-producing *E*. *coli* of the ST641 and non-ESBL producing *E*. *coli* of ST1210 were isolated from humans in a Spanish hospital between 2004 and 2005 [41]. The comparison to literature indicated that several of the detected *E*. *coli* occurred also in human population.

For some *E*. *coli* isolates we obtained positive growth on CHROMagar STEC but negative results in PCR based SLT-1 and SLT-2 gene detection. This was also reported in other studies [42,43,44]. Hirvonen and colleagues [43] pointed out, that a growth on CHROMagar STEC is highly correlated with a tellurite resistance gene (*terD*) which might have been an additional target, but was not applied here.

None of the tested *E*. *coli* isolates of this study showed a carbapeneme resistance. Currently only few data are available of the spread of carbapenemase containing *E*. *coli* in livestock husbandry in German. A first description of a carbapenem resistant *E*. *coli* isolated from a German pig fattening husbandry in 2011 was given by Fischer and colleagues [45]. A carbapenem resistant ESBL-producing *E*. *coli* was furthermore detected in samples of a beef cattle farm [19] and in horse, cat and dog [23]. Because carbapenem antibiotics are not allowed for the application in the veterinarian field in Germany currently only few carbapenem resistant *E*. *coli* originating from livestock husbandry were detected so far.

## Conclusions

In summary our study showed the presence of a clonally diverse number of ESBL-producing *E*. *coli* strains in manure samples originating from livestock husbandry which can still be detected in output samples of biogas plants and thereby released into the environment. Our study clearly showed that biogas plant processes can reduce but not eliminate ESBL-producing *E*. *coli* from livestock husbandry. We furthermore clearly showed that the application of a selective pre-enrichment procedures is a pre-requisite of an efficient detection of ESBL-producing *E*. *coli* especially from output samples of biogas plants. The applied CHROMagar ESBL is not selective for growth of ESBL-producing *E*. *coli* but *E*. *coli* can be specifically detected as pink colonies. The combined method of pre-enrichment and cultivation on CHROMagar ESBL can be applied as a fast first step detection method for the presence of ESBL-producing *E*. *coli* in input and output samples of biogas plants. Comparative genotypic analysis including BOX-fingerprinting, MLST analysis, *E*. *coli* phylogrouping and resistance gene characterization is necessary to investigated the distribution pattern of specific *E*. *coli* populations. This combined approach enabled us to illustrate for ESBL producing *E*. *coli* of ST410 and ST58 a direct transfer from manure through biogas plants.

## Materials and Methods

### Biogas plants

Input and output samples of five German biogas plants (BGA 001, 002, 005, 006, 012) were investigated between May and October 2012 and of two biogas plants (BGA 001 and BGA 015) more intensively between February 2013 and February 2014. The investigated biogas plants were located in three regions in Germany, in the north of Hesse (BGA 001, 002, 005, and 006), in Bremen (BGA 012) and in Bavarian near Munich (BGA 015). In Hesse, biogas plants show a mean distance of approximately 10 km. All biogas plants were mesophilic, except BGA 012 was thermophilic. Input material (manure sources), bioenergy crops supplemented to the main digester and process conditions of the different biogas plants are listed in S1 Table.

### Sampling

During the 2012 sampling for each of the 6 investigated biogas plants one input and one output samples were taken at the same day. In 2013/2014 the two investigated biogas plants were investigated four time in an annual cycle. Output samples were thereby sampled 8–10 weeks after the respective input samples (input samples were taken in February, April, July/August and October 2013, respective output samples in April, July/August, October 2013 and February 2014). Input samples consisted of slurry and solid manure compounds mixed individually for each biogas plant in the ration as added to biogas digester (excluding bioenergy crops). The mean water content was 89.2% (S1 Table). Output samples were obtained from premixed post fermenters by randomly sampling within one hour; at all 30 L sample material from a continuously mixed post fermenter tank by taking 5 L sample every 10 minutes. The mean water content was 91.3%. Samples for microbiological analysis (200 mL subsamples) were collected in 250 mL sterile PE bottles and stored immediately at 6°C, transported into the lab and processed at the same day. Fresh liquid samples (containing some solid straw and manure particles in the input sample) were used for analysis. No specific permissions were required for these locations/activities and sampling was done in assent with the farmers.

### Direct cultivation on CHROMagar ESBL (enumeration of ESBL-producer)

For the enumeration of ESBL-producing *E*. *coli* colony forming units (CFUs) were counted on CHROMagar ESBL (CHROMagar, Paris, France). Bacteria were detached from 10 g fresh input or output samples by shaking the samples for 5 min in 100 mL filter-sterilized 0.2 (w/v)% tetra-sodium-pyrophosphate (TSPP) buffer in autoclaved 250 mL SCHOTT bottles on a horizontal shaker (150 rpm) at room temperature in the dark. To remove larger particles of the samples before analysis samples were in a second step incubated for 30 min without shaking. From the upper part of the supernatant 40 mL were used for subsequent analyses. A serial dilution was generated in autoclaved 0.9% (v/w) NaCl (10^0^ to 10^-3^) and 100 μL of each dilution step were plated in triplicates on CHROMagar ESBL. Agar plates were incubated for 48 h at 37°C in the dark. Based on the leaflet of the company dark pink colonies were counted as potential ESBL-producing *E*. *coli*, metallic blue colonies as ESBL-producing *Klebsiella*, *Enterobacter*, and *Citrobacter* and brown coloured colonies as ESBL-producing *Proteus*. Colony forming units (CFUs) were calculated per gram dry weight of input and output samples.

### ESBL-producer specific pre-enrichment procedures

A selective pre-enrichment was applied to enrich ESBL-producing *E*. *coli*. Therefore, 0.1, 1, and 10 g fresh input or output samples were incubated directly in 0.9, 9, or 90 mL LB-broth containing 0.5 mg L^-1^ ceftazidim (CAZ) and 0.5 mg L^-1^ cefotaxim (CTX). All incubations were done in triplicates. After 24 h of incubation at 37°C and 180 rpm, 10 μL of the pre-enrichment cultures were streaked on CHROMagar ESBL using a 13-line purification streaking method. Plates were incubated for 24 h at 37°C. Single pink and blue colonies were screened for ESBL genes using the ESBL-gene detecting multiplex-PCR as described [46]. An overview of the procedure is given in Fig. 2.

### Isolation and maintenance of isolates

Most abundant colonies grown on CHROMagar ESBL plates were picked and purified using several transfer steps of single colonies. First purification steps were performed on CHROMagar ESBL at 37°C. For sub-cultivation and maintenance isolates were cultured on LB-agar containing 0.5 mg L^-1^ CAZ and 0.5 mg L^-1^ CTX. Purity of the isolates was checked by gram-staining and microscopy. For long-term preservation fresh biomass of the isolates was suspended in calf serum albumin (Micronics) and stored at -20°C or -80°C.

### Growth tests on CHROMagar KPC and CHROMagar STEC

All isolates identified as ESBL-producing *E*. *coli* were tested for growth on CHROMagar KPC and CHROMagar STEC (both CHROMagar, Paris, France). Growth and colony pigmentation were monitored after 24 h of incubation at 37°C.

### Generation of bacterial cell lysate for molecular biological analysis

For molecular analysis of isolates two loops of biomass were washed in 500 μL sterile sodium phosphate puffer (120 mM, pH 8.0), pelleted by centrifugation for 2 min at 13780 g (RT) and suspended in 500 μL DNase and RNase free water (Invitrogen, Germany). DNA was released from the cells by three cycles of freezing (-20°C) and headed to 100°C for 1 min 30 seconds in a heating block. Cell lysates were stored at -20°C; 1–2 μL of the supernatant was used as template for PCR amplifications.

### Phylogenetic identification of isolates by partial 16S rRNA gene sequencing

For phylogenetically identification of isolates partial 16S rRNA gene fragments were amplified with primer 27F and 1492R [47]. PCRs were performed in a total volume of 25 μL including 1x buffer, 200 μM of each dNTPs, 0.2 μM of each primer, 0.2 mg mL^-1^ BSA, 0.02 U Dream *Taq* DNA polymerase (all chemicals except primers from Fermentas/ Thermo Scientific). Cycle conditions were as followed: 95°C for 3 min followed by 30 cycles of 95°C for 30 sec, 54°C for 30 sec, and 72°C for 1 min 30 sec, and finally 72°C for 7 min. Sanger sequencing was performed with the forward primer 27F. Sequences were corrected manually in MEGA 5 [48] based on the electropherograms by removing unclear 5´ and 3´ ends of the sequences. For phylogenetic identification closest phylogenetic related type strains were determined by BLAST analysis using the EzTaxon-e Database [49].

### Screening for beta-lactamase genes

Isolates were screened for the presence of *beta-lactamase genes (bla)* genes using the Multiplex-PCR assay published by Monstein and colleagues [46] targeting *bla*_CTX-M_, *bla*_TEM_ and *bla*_SHV_ genes. PCR reactions were performed in a total volume of 10 μL with PCR reagents as described above, except 0.4 μM of each primer and an annealing temperature (Ta) of 60°C and an elongation step of 2 min. The amplification of *resistance* genes, *bla*_SHV_: 747 bp, *bla*_CTX-M_: 593 bp, *bla*_TEM_: 445 bp, was controlled by 1% agarose electrophoresis. Carbapenemase gene screening was performed by real-time PCR based multiplex-PCR system as described by Monteiro and colleagues [50] detecting *bla*_KPC_, *bla*_NDM_, *bla*_GES_, *bla*_OXA-48_, *bla*_IMP_, and bla_VIM_ genes in parallel using the Sso Fast Eva Green Realtime PCR mastermix (Bio-Rad). PCRs were performed in a total volume of 10 μl with 0.2 μM of each primer (except 1.2 μM for IMP primers) using a CFX96 Touch cycler (Bio-Rad) and following cycle conditions: 98°C for 2 min, followed by 35 cycles of 98°C for 20 sec, 55°C for 20 sec, and 72°C for 20 sec. Melting-curve analysis was subsequently performed by heating from 65°C to 95°C with 0.5°C/ 5 sec to identify the different carbapenemase genes based on the melt peaks as describe by Monteiro et al. [50]. PCR products were controlled by agarose gel electrophoresis. If positive signals were obtained the presence of respective genes was proven by using single primer systems for amplification of *E*. *coli* strains carrying a *bla*_CTX-M-14_ and *bla*_TEM-20_ (provided by Prof. Dr. U. Rösler, Freie Universität Berlin, Germany), *E*. *coli* DSM 22311 carrying a *bla*_SHV_ gene, *Klebsiella pneumoniae* strains carrying carbapenemase genes, strains 50/11 (NDM-1), 234/11 (KPC-2) and 229/09 (OXA-48) and *E*. *coli* 188/11 carrying a VIM-1 gene (provided by Prof. Dr. Christa Ewers, Justus Liebig University Giessen) were used as amplification controls.

### *Bla*_CTX-M_, *bla*_TEM_ and *bla*_SHV_ gene characterization

*Bla*_CTX-M_-genes were assigned to different CTX-M-groups using group specific primer systems [51] for the detection of CTX-M-1 and 9 group genes and [52] for the detection of CTX-M-8 group genes (CTXMgp8-F, CTXMgp8-R) and sequenced with the respective primer systems. *Bla*_CTX-M_ genes not amplified with those primer systems were amplified and sequence with the universal *bla*_CTX-M_ primer system [51] or with the CTX-M primer system used in the multiplex-PCR [46]. Partial *bla*_TEM_ genes were also amplified with the TEM primers applied for multiplex-PCR [46] or to obtain longer gene fragments with universal TEM primer as described by Grimm et al. [53]. *Bla*_SHV_ genes were PCR amplified and sequence with universal primers covering the nearly full-length gene as described by Pai et al. [54]. Sequence analysis was always carried out with respective forward primer. Phylogenetic analysis were performed in MEGA 5. Sequences were corrected manually based on electropherograms. Open reading frame correct alignments were performed by using full-length reference genes obtained from GenBank (http://www.ncbi.nlm.nih.gov/entrez/query) and *bla*_CTX-M_, *bla*_TEM_, and *bla*_SHV_ database (http://www.lahey.org/Studies/). Nucleotide sequences were translated into amino acid sequences and aligned using ClustalW implemented in MEGA5. To assign partially sequenced genes to *bla*_CTX-M_, *bla*_TEM_ and *bla*_SHV_ subtypes phylogenetic trees were calculated based on amino acid sequences using the maximum-likelihood method and the Jones Taylor Thornton model and 100 bootstrap. Subtypes of the CTX-M-1 group were further differentiated by using differences in selected amino acids, *bla*_CTX-M-1_, *bla*_CTX-M-15_, *bla*_CTX-M-32_ were differentiated based on three to four amino acids (aa117, aa143, and aa242). Within the CTX-M-9 group *bla*_CTX-M-9_ and *bla*_CTX-M-14_ were differentiated based on the amino acid 234 (*bla*_CTX-M-9_: alanine; *bla*_CTX-M-14_: valine).

### Screening for Shiga like toxin (SLT) genes

*E*. *coli* isolates were screened for the presence of SLT genes using a modified STEC multiplex PCR based on the system described by Cebula and colleagues [55] including the primer systems for SLT I, SLT II, and β-glucuronidase (uidA). In addition, an universal 16S rRNA gene targeting primer system was added as internal amplification control (16Sup1/16Sup2, [56]). PCR reactions were set up in a total volume of 10 μL using the DreamTaq DNA polymerase as described above, except 0.5 μM of each primer, a Ta of 64°C, and 1 min elongation. Amplification products were checked on 1.7% agarose gels.

### *E*. *coli* phylo-grouping multiplex-PCR

*E*. *coli* isolated where assigned to the four major phylogenetic *E*. *coli* groups A, B1, B2 and D using the multiplex PCR approach [57]. Modified PCR conditions were applied, adjusted to a volume of 10 μL PCR and DreamTaq DNA polymerase standard conditions as described above using 0.125 μM of each primer according to [57], a primer annealing temperature of 65°C and 30 sec for elongation. PCR products were separated by 1.5% agarose gel electrophoresis and *E*. *coli* isolates were assigned to phylogenetic groups using a dichotomous decision tree [10].

### Genotypic differentiation of *E*. *coli* isolates using BOX-PCR (molecular fingerprinting)

ESBL-producing *E*. *coli* isolates were differentiated at the genomic level by BOX-PCR using primer BOX1A [58] as described previously [59]. Cluster analysis of BOX-PCR pattern was performed in GelCompar II (Applied Maths) using the UPGMA clustering based on the Pearson correlation.

### Multilocus sequence typing (MLST)

MLST sequence types were determined for genetically different ESBL-producing *E*. *coli* isolates using the *E*. *coli* MLST scheme based on the 7 housekeeping genes *adk*, *fumC*, *gyrB*, *icd*, *mdh*, *purA* and *recA* [60]. Primer and amplification conditions were used according to Wirth et al. [60] except primer recAR was used as reverse primer for recA amplification and (http://mlst.ucc.ie/mlst/dbs/Ecoli/documents/primersColi_html) and primer icd-P1(-79) [61] used as forward primer for icd amplification. Reverse primers were used for Sanger sequencing. Sequences were corrected manually based on the electropherograms and trimmed to the gene fragments necessary for ST analysis using MEGA5 and sequence types (ST) were identified using the MLST database (http://mlst.ucc.ie/mlst/dbs/Ecoli/).

### Antimicrobial susceptibility testing

Susceptibility testing of *E*. *coli* isolates carrying ESBL genes to ten veterinary relevant antibiotics was tested for a selection for using a standard sensitivity 96 well plate test panel (Micronaut S, Merlin, Bornheim-Hersel) to determine minimal inhibitory concentrations (MIC) values. Isolate preparation and treatment was done accordingly to CLSI guidelines [62]. For each antibiotic a concentration range of eight concentrations was tested [values in μg mL^-1^] for: amoxicillin (0.5–64), cefquinom (0.25–32) (+/- clavulanic acid), ceftiofur (0.25–32) (+/- clavulanic acid), enrofloxacin (0.0625–8), florfenicol (0.5–64), oxacillin (0.25–32), sulfamethoxazole (4–256), tetracyclin (0.25–32), trimethoprim/sulfamethoxazole (0.0625/1.125–8/125), and tylosin (0.125–16). Meropenem was tested accordingly in a 96 well plate. Tested concentrations ranged from 0.0625 to 64 mg L^-1^. Meropenem was directly adjusted to the growth medium in test panel wells.

### Nucleotide sequence deposition

The GenBank/EMBL/DDBJ accession number for 16S rRNA gene sequences of bacterial isolates are KJ831392 to KJ831561 and KM658573 to KM658586; for CTX-M coding genes KJ918507 to KJ918509; KJ918513 to KJ918537, and KM658587 to KM658595; for SHV coding genes KP165080 to KP165082, and gene sequences obtained from housekeeping genes of *E*. *coli* isolates were partially published under KJ918538 to KJ918564 (*adk*), KJ918565 to KJ918591 (*fumC*), KJ918592 to KJ918618 (*gyrB*), KJ918619 to KJ918645(*icd*), KJ918700 to KJ918726 (*mdh*), KJ918646 to KJ918672 (*purA*), and KJ918673 to KJ918699 (*recA*).

## Supporting Information

S1 Fig Overview of growth on CHROMagar ESBL after direct plating (sample dilutions 10^0^ to 10^-2^) and after pre-enrichment of 0.1, 1, and 10 g. (PDF)

S2 Fig Distribution of *E*. *coli* genotypes (defined by BOX-PCR pattern) in input and output samples of the investigated biogas plants. (PDF)

S3 Fig Phylogenetic assignment of CTX-M genes detected in ESBL isolates to main CTX-M groups based on partial amino acid sequence based phylogenetic analysis. (PDF)

S4 Fig Multilocus sequence based maximum-likelihood tree based on concatenated nucleotide sequences showing the phylogenetic variety of *E*. *coli* isolates. (PDF)

S1 Table Overview of the composition of input samples and bioenergy crops from the different biogas plants investigated in 2012 (A) and 2013 (B). (PDF)

## References

[1] Friese A, Schulz J, Laube H, von Salviati C, Hartung J, Roesler U. Faecal occurrence and emissions of livestock-associated methicillin-resistant Staphylococcus aureus (laMRSA) and ESBL/AmpC-producing E. *coli* from animal farms in Germany. Berl Munch Tierarztl Wochenschr 2013;126(3–4): 175–180. 23540202

[2] Hartmann A, Locatelli A, Amoureux L, Depret G, Jolivet C, Gueneau E, et al Occurrence of CTX-M producing Escherichia coli in soils, cattle, and farm environment in France (Burgundy Region). Front Microbiol 2012;3: 83 10.3389/fmicb.2012.00083 22408639 PMC3297819

[3] Snow LC, Warner RG, Cheney T, Wearing H, Stokes M, Harris K, et al Risk factors associated with extended spectrum beta-lactamase *Escherichia coli* (CTX-M) on dairy farms in North West England and North Wales. Prev Vet Med 2012;106(3–4): 225–234. 10.1016/j.prevetmed.2012.04.008 22552330

[4] Ewers C, Bethe A, Semmler T, Guenther S, Wieler LH. Extended-spectrum beta-lactamase-producing and AmpC-producing Escherichia coli from livestock and companion animals, and their putative impact on public health: a global perspective. Clin Microbiol Infect 2012;18(7): 646–655. 10.1111/j.1469-0691.2012.03850.x 22519858

[5] Heuer H, Schmitt H, Smalla K. Antibiotic resistance gene spread due to manure application on agricultural fields. Curr Opin Microbiol 2011;14(3): 236–243. 10.1016/j.mib.2011.04.009 21546307

[6] Weiland P. Biogas production: current state and perspectives. Appl Microbiol Biotechnol 2010;85(4): 849–860. 10.1007/s00253-009-2246-7 19777226

[7] Angelidaki I, Ellegaard L, Ahring BK. A mathematical model for dynamic simulation of anaerobic digestion of complex substrates: focusing on ammonia inhibition. Biotechnol Bioeng 1993;42(2): 159–166. 18612976 10.1002/bit.260420203

[8] Philipp W, Hölzle LE. Krankheitskeime in Gärresten aus Biogasanlagen? Gefahrstoffe-Reinhaltung der Luft 2012;72(5): 216–220.

[9] Resende JA, Silva VL, de Oliveira TL, de Oliveira Fortunato S, da Costa Carneiro J, Otenio MH, et al Prevalence and persistence of potentially pathogenic and antibiotic resistant bacteria during anaerobic digestion treatment of cattle manure. Bioresour Technol 2014;153: 284–291. 10.1016/j.biortech.2013.12.007 24374028

[10] Clermont O, Bonacorsi S, Bingen E. Rapid and simple determination of the Escherichia coli phylogenetic group. Appl Environ Microbiol 2000;66(10): 4555–4558. 11010916 10.1128/aem.66.10.4555-4558.2000 PMC92342

[11] Higgins CS, Avison MB, Jamieson L, Simm AM, Bennett PM, Walsh TR. Characterization, cloning and sequence analysis of the inducible Ochrobactrum anthropi AmpC beta-lactamase. J Antimicrob Chemother 2001;47(6): 745–754. 11389106 10.1093/jac/47.6.745

[12] Nadjar D, Labia R, Cerceau C, Bizet C, Philippon A, Arlet G. Molecular characterization of chromosomal class C β-lactamase and its regulatory gene in Ochrobactrum anthropi. Antimicrob Agents Chemother 2001;45(8): 2324–2330. 11451692 10.1128/AAC.45.8.2324-2330.2001 PMC90649

[13] Lister PD, Wolter DJ, Hanson ND. Antibacterial-resistant Pseudomonas aeruginosa: clinical impact and complex regulation of chromosomally encoded resistance mechanisms. Clin Microbiol Rev. 2009 10;22(4): 582–610. 10.1128/CMR.00040-09 19822890 PMC2772362

[14] Perez F, Hujer AM, Hujer KM, Decker BK, Rather PN, Bonomo RA. Global challenge of multidrug-resistant Acinetobacter baumannii. Antimicrob Agents Chemother. 2007;51(10): 3471–3484. 17646423 10.1128/AAC.01464-06 PMC2043292

[15] Grohs P, Tillecovidin B, Caumont-Prim A, Carbonnelle E, Day N, Podglajen I, et al Comparison of five media for detection of extended-spectrum Beta-lactamase by use of the wasp instrument for automated specimen processing. J Clin Microbiol. 2013;51(8): 2713–2716. 10.1128/JCM.00077-13 23698524 PMC3719624

[16] Saito R, Koyano S, Nagai R, Okamura N, Moriya K, Koike K. Evaluation of a chromogenic agar medium for the detection of extended-spectrum beta-lactamase-producing Enterobacteriaceae. Lett Appl Microbiol. 2010;51(6): 704–706. 21117288 10.1111/j.1472-765x.2010.02945.x

[17] Randall LP, Kirchner M, Teale CJ, Coldham NG, Liebana E, Clifton-Hadley F. Evaluation of CHROMagar CTX, a novel medium for isolating CTX-M-ESBL-positive Enterobacteriaceae while inhibiting AmpC-producing strains. J Antimicrob Chemother. 2009;63(2): 302–308. 10.1093/jac/dkn485 19043079

[18] Laube H, Friese A, von Salviati C, Guerra B, Kasbohrer A, Kreienbrock L, et al Longitudinal Monitoring of Extended-Spectrum-Beta-Lactamase/AmpC-Producing Escherichia coli at German Broiler Chicken Fattening Farms. Appl Environ Microbiol. 2013;79(16): 4815–4820. 10.1128/AEM.00856-13 23747697 PMC3754693

[19] Schmid A, Hormansdorfer S, Messelhäusser U, Käsbohrer A, Sauter-Louis C, Mansfeld R. Prevalence of extended-spectrum beta-lactamase-producing Escherichia coli on bavarian dairy and beef cattle farms. Appl Environ Microbiol. 2013;79(9): 3027–3032. 10.1128/AEM.00204-13 23455336 PMC3623142

[20] Kola A, Kohler C, Pfeifer Y, Schwab F, Kuhn K, Schulz K, et al High prevalence of extended-spectrum-beta-lactamase-producing *Enterobacteriaceae* in organic and conventional retail chicken meat, Germany. J of Antimicrob Chemoth. 2012;67(11): 2631–2634. 10.1093/jac/dks295 22868643

[21] Boxall ABA, Fogg LA, Blackwell PA, Kay P, Pemberton EJ, Croxford A. Veterinary medicines in the environment. Reviews of Environmental Contamination and Toxicology. 2004;180: 1–91. 14561076 10.1007/0-387-21729-0_1

[22] Tenaillon O, Skurnik D, Picard B, Denamur E. The population genetics of commensal Escherichia coli. Nat Rev Microbiol. 2010;8(3): 207–217. 10.1038/nrmicro2298 20157339

[23] Schmiedel J, Falgenhauer L, Domann E, Bauerfeind R, Prenger-Berninghoff E, Imirzalioglu C, et al Multiresistant extended-spectrum beta-lactamase-producing Enterobacteriaceae from humans, companion animals and horses in central Hesse, Germany. BMC Microbiol. 2014;14: 187. 25014994 10.1186/1471-2180-14-187 PMC4105247

[24] Günther S, Bethe A, Fruth A, Semmler T, Ulrich RG, Wieler LH, et al Frequent combination of antimicrobial multiresistance and extraintestinal pathogenicity in *Escherichia coli* isolates from urban rats (*Rattus norvegicus*) in Berlin, Germany. PLoS One. 2012;7(11): e50331 10.1371/journal.pone.0050331 23189197 PMC3506595

[25] Jakobsen L, Garneau P, Kurbasic A, Bruant G, Stegger M, Harel J, et al Microarray-based detection of extended virulence and antimicrobial resistance gene profiles in phylogroup B2 Escherichia coli of human, meat and animal origin. J Med Microbiol. 2011;60(Pt 10): 1502–1511. 10.1099/jmm.0.033993-0 21617024

[26] Manoharan A, Premalatha K, Chatterjee S, Mathai D. Correlation of TEM, SHV and CTX-M extended-spectrum beta lactamases among Enterobacteriaceae with their in vitro antimicrobial susceptibility. Indian J Med Microbiol. 2011;29(2): 161–164. 10.4103/0255-0857.81799 21654112

[27] Kanamori H, Navarro RB, Yano H, Sombrero LT, Capeding MR, Lupisan SP, et al Molecular characteristics of extended-spectrum beta-lactamases in clinical isolates of Enterobacteriaceae from the Philippines. Acta Trop. 2011;120(1–2): 140–145. 21820398 10.1016/j.actatropica.2011.07.007

[28] Singh A, Shahid M, Sobia F, Umesh, Khan HM. Comparative study on occurrence of class A and class C β-lactamase genes and their co-occurrence in Indian Enterobacteriaceae during years 2009 and 2010. Asian Pac J Trop Med. 2011;4(10): 764–768. 10.1016/S1995-7645(11)60190-9 22014729

[29] Kaur M, Aggarwal A. Occurrence of the CTX-M, SHV and the TEM Genes Among the Extended Spectrum beta-Lactamase Producing Isolates of Enterobacteriaceae in a Tertiary Care Hospital of North India. J Clin Diagn Res. 2013;7(4): 642–645. 10.7860/JCDR/2013/5081.2872 23730637 PMC3644435

[30] Valenza G, Nickel S, Pfeifer Y, Eller C, Krupa E, Lehner-Reindl V, et al Extended-spectrum-beta-lactamase-producing Escherichia coli as intestinal colonizers in the German community. Antimicrob Agents Chemother. 2014;58(2):1228–1230. 10.1128/AAC.01993-13 24295972 PMC3910888

[31] Leistner R, Meyer E, Gastmeier P, Pfeifer Y, Eller C, Dem P, et al Risk factors associated with the community-acquired colonization of extended-spectrum beta-lactamase (ESBL) positive Escherichia coli. An exploratory case-control study. PLoS One. 2013;8(9): e74323 10.1371/journal.pone.0074323 24040229 PMC3770595

[32] Blaak H, Hamidjaja RA, van Hoek AH, de Heer L, de Roda Husman AM, Schets FM. Detection of extended-spectrum beta-lactamase (ESBL)-producing Escherichia coli on flies at poultry farms. Appl Environ Microbiol. 2014;80(1): 239–246. 10.1128/AEM.02616-13 24162567 PMC3910986

[33] Vincent C, Boerlin P, Daignault D, Dozois CM, Dutil L, Galanakis C, et al Food reservoir for Escherichia coli causing urinary tract infections. Emerg Infect Dis. 2010;16(1): 88–95. 10.3201/eid1601.091118 20031048 PMC2874376

[34] Rogers BA, Sidjabat HE, Paterson DL. Escherichia coli O25b-ST131: a pandemic, multiresistant, community-associated strain. J Antimicrob Chemother. 2011;66(1): 1–14. 10.1093/jac/dkq415 21081548

[35] Hernandez J, Johansson A, Stedt J, Bengtsson S, Porczak A, Granholm S, et al Characterization and comparison of extended-spectrum beta-lactamase (ESBL) resistance genotypes and population structure of Escherichia coli isolated from Franklin's gulls (Leucophaeus pipixcan) and humans in Chile. PLoS One. 2013;8(9): e76150 10.1371/journal.pone.0076150 24098774 PMC3786981

[36] Dobiasova H, Dolejska M, Jamborova I, Brhelova E, Blazkova L, Papousek I, et al Extended spectrum beta-lactamase and fluoroquinolone resistance genes and plasmids among Escherichia coli isolates from zoo animals, Czech Republic. FEMS Microbiol Ecol. 2013;85(3): 604–611. 10.1111/1574-6941.12149 23679004

[37] Ewers C. Extended-spectrum beta-lactamases producing Gram-negative bacteria in companion animals: action is clearly warranted! Berl Munch Tierarztl Wochenschr. 2011;124: 94–101. 21462862

[38] Huber H, Zweifel C, Wittenbrink MM, Stephan R. ESBL-producing uropathogenic Escherichia coli isolated from dogs and cats in Switzerland. Vet Microbiol. 2013 23;162(2–4): 992–996. 23177909 10.1016/j.vetmic.2012.10.029

[39] Schink AK, Kadlec K, Schwarz S. Analysis of bla(CTX-M)-carrying plasmids from Escherichia coli isolates collected in the BfT-GermVet study. Appl Environ Microbiol. 2011;77(20): 7142–7146. 10.1128/AEM.00559-11 21685166 PMC3194854

[40] Mellmann A, Bielaszewska M, Kock R, Friedrich AW, Fruth A, Middendorf B, et al Analysis of collection of hemolytic uremic syndrome-associated enterohemorrhagic Escherichia coli. Emerg Infect Dis. 2008;14(8): 1287–1290. 10.3201/eid1408.071082 18680658 PMC2600372

[41] Ruiz del Castillo B, Vinue L, Roman EJ, Guerra B, Carattoli A, Torres C, et al Molecular characterization of multiresistant Escherichia coli producing or not extended-spectrum beta-lactamases. BMC Microbiol. 2013;13:84 10.1186/1471-2180-13-84 23586437 PMC3637601

[42] Gouali M, Ruckly C, Carle I, Lejay-Collin M, Weill FX. Evaluation of CHROMagar STEC and STEC O104 chromogenic agar media for detection of Shiga Toxin-producing Escherichia coli in stool specimens. J Clin Microbiol. 2013;51(3): 894–900. 10.1128/JCM.03121-12 23284030 PMC3592037

[43] Hirvonen JJ, Siitonen A, Kaukoranta SS. Usability and performance of CHROMagar STEC medium in detection of Shiga toxin-producing Escherichia coli strains. J Clin Microbiol. 2012;50(11): 3586–3590. 10.1128/JCM.01754-12 22933601 PMC3486272

[44] Tzschoppe M, Martin A, Beutin L. A rapid procedure for the detection and isolation of enterohaemorrhagic Escherichia coli (EHEC) serogroup O26, O103, O111, O118, O121, O145 and O157 strains and the aggregative EHEC O104:H4 strain from ready-to-eat vegetables. Int J Food Microbiol. 2012;152(1–2): 19–30. 10.1016/j.ijfoodmicro.2011.09.010 22071287

[45] Fischer J, Rodriguez I, Schmoger S, Friese A, Roesler U, Helmuth R, et al Escherichia coli producing VIM-1 carbapenemase isolated on a pig farm. J Antimicrob Chemother. 2012;67(7): 1793–1795. 10.1093/jac/dks108 22454489

[46] Monstein HJ, Ostholm-Balkhed A, Nilsson MV, Nilsson M, Dornbusch K, Nilsson LE. Multiplex PCR amplification assay for the detection of blaSHV, blaTEM and blaCTX-M genes in Enterobacteriaceae. Apmis. 2007;115(12): 1400–1408. 10.1111/j.1600-0463.2007.00722.x 18184411

[47] Lane DJ. 16S/23S rRNA sequencing nucleic acid techniques in bacterial systematics In: Stackebrandt E, Goodfellow M, editors. Nucleic acid techniques in bacterial systematics. Wiley, Chichester; 1991 pp. 115–175

[48] Tamura K, Peterson D, Peterson N, Stecher G, Nei M, Kumar S. MEGA5: molecular evolutionary genetics analysis using maximum likelihood, evolutionary distance, and maximum parsimony methods. Mol Biol Evol. 2011;28(10): 2731–2739. 10.1093/molbev/msr121 21546353 PMC3203626

[49] Kim OS, Cho YJ, Lee K, Yoon SH, Kim M, Na H, et al Introducing EzTaxon-e: a prokaryotic 16S rRNA gene sequence database with phylotypes that represent uncultured species. Int J Syst Evol Microbiol. 2012;62(Pt 3): 716–721. 10.1099/ijs.0.038075-0 22140171

[50] Monteiro J, Widen RH, Pignatari AC, Kubasek C, Silbert S. Rapid detection of carbapenemase genes by multiplex real-time PCR. J Antimicrob Chemother. 2012;67(4): 906–909. 10.1093/jac/dkr563 22232516

[51] Batchelor M, Hopkins K, Threlfall EJ, Clifton-Hadley FA, Stallwood AD, Davies RH, et al bla(CTX-M) genes in clinical Salmonella isolates recovered from humans in England and Wales from 1992 to 2003. Antimicrob Agents and Chemotherapy. 2005;49(4): 1319–1322. 15793104 10.1128/AAC.49.4.1319-1322.2005 PMC1068621

[52] Hopkins KL, Deheer-Graham A, Threlfall EJ, Batchelor MJ, Liebana E. Novel plasmid-mediated CTX-M-8 subgroup extended-spectrum beta-lactamase (CTX-M-40) isolated in the UK. Int J Antimicrob Agents. 2006;27(6):572–575. 16697557 10.1016/j.ijantimicag.2006.03.003

[53] Grimm V, Ezaki S, Susa M, Knabbe C, Schmid RD, Bachmann TT. Use of DNA microarrays for rapid genotyping of TEM beta-lactamases that confer resistance. J Clin Microbiol. 2004;42(8): 3766–3774. 15297528 10.1128/JCM.42.8.3766-3774.2004 PMC497576

[54] Pai H, Lyu S, Lee JH, Kim J, Kwon Y, Kim JW, et al Survey of extended-spectrum beta-lactamases in clinical isolates of Escherichia coli and Klebsiella pneumoniae: prevalence of TEM-52 in Korea. J Clin Microbiol. 1999;37(6): 1758–1763. 10325320 10.1128/jcm.37.6.1758-1763.1999 PMC84943

[55] Cebula TA, Payne WL, Feng P. Simultaneous identification of strains of Escherichia coli serotype O157:H7 and their Shiga-like toxin type by mismatch amplification mutation assay-multiplex PCR. J Clin Microbiol. 1995;33(1): 248–250. 7535315 10.1128/jcm.33.1.248-250.1995 PMC227922

[56] Poulsen AB, Skov R, Pallesen LV. Detection of methicillin resistance in coagulase-negative staphylococci and in staphylococci directly from simulated blood cultures using the EVIGENE MRSA Detection Kit. J Antimicrob Chemother. 2003;51(2): 419–421. 12562714 10.1093/jac/dkg084

[57] Doumith M, Day MJ, Hope R, Wain J, Woodford N. Improved multiplex PCR strategy for rapid assignment of the four major Escherichia coli phylogenetic groups. J Clin Microbiol. 2012;50(9): 3108–3110. 10.1128/JCM.01468-12 22785193 PMC3421818

[58] Versalovic J, Schneider M, de Bruijn FJ, Lupski JR. Genomic fingerprinting of bacteria using repetitive sequence-based polymerase chain reaction. Methods Mol Cell Biol. 1994;5: 25–40.

[59] Glaeser SP, Galatis H, Martin K, Kämpfer P. Niabella hirudinis and Niabella drilacis sp. nov., isolated from the medicinal leech Hirudo verbana. Int J Syst Evol Microbiol. 2013;63(Pt 9): 3487–3493. 10.1099/ijs.0.050823-0 23543503

[60] Wirth T, Falush D, Lan R, Colles F, Mensa P, Wieler LH, et al Sex and virulence in Escherichia coli: an evolutionary perspective. Mol Microbiol. 2006;60(5): 1136–1151. 16689791 10.1111/j.1365-2958.2006.05172.x PMC1557465

[61] Bielaszewska M, Prager R, Kock R, Mellmann A, Zhang W, Tschape H, et al Shiga toxin gene loss and transfer in vitro and in vivo during enterohemorrhagic Escherichia coli O26 infection in humans. Appl. Environ. Microbiol. 2007;73(10): 3144–3150. 17400784 10.1128/AEM.02937-06 PMC1907125

[62] Clinical and Laboratory Standards Institute Methods for Dilution Antimicrobial Susceptibility Tests for Bacteria That Grow Aerobically; Approved Standard-Ninth Edition, M07-A9. CLSI, Wayne, PA, USA; 2012

