# Supplementary material for: Improved Detection of Extended Spectrum Beta-Lactamase (ESBL)-Producing Escherichia coli in Input and Output Samples of German Biogas Plants by a Selective Pre-Enrichment Procedure
Source: PLoS One. 2015 Mar 23;10(3):e0119791. doi: 10.1371/journal.pone.0119791 (PMC4370489; doi:10.1371/journal.pone.0119791)
Supplement: S1 Fig — (PDF) [file pone.0119791.s001.pdf]

BGA 001

a b c

Direct plating

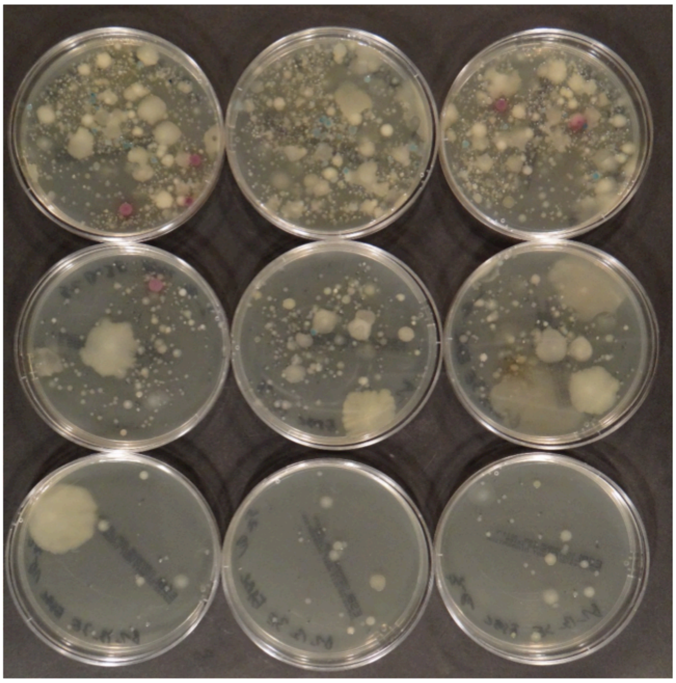

10^0

10^{-1}

10^{-2}

a b c

Pre enrichment

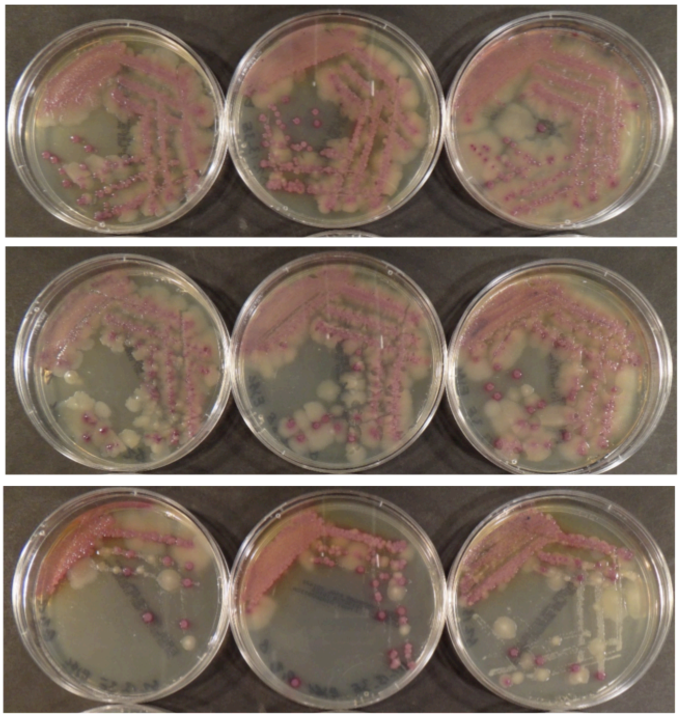

10 g

1 g

0.1 g

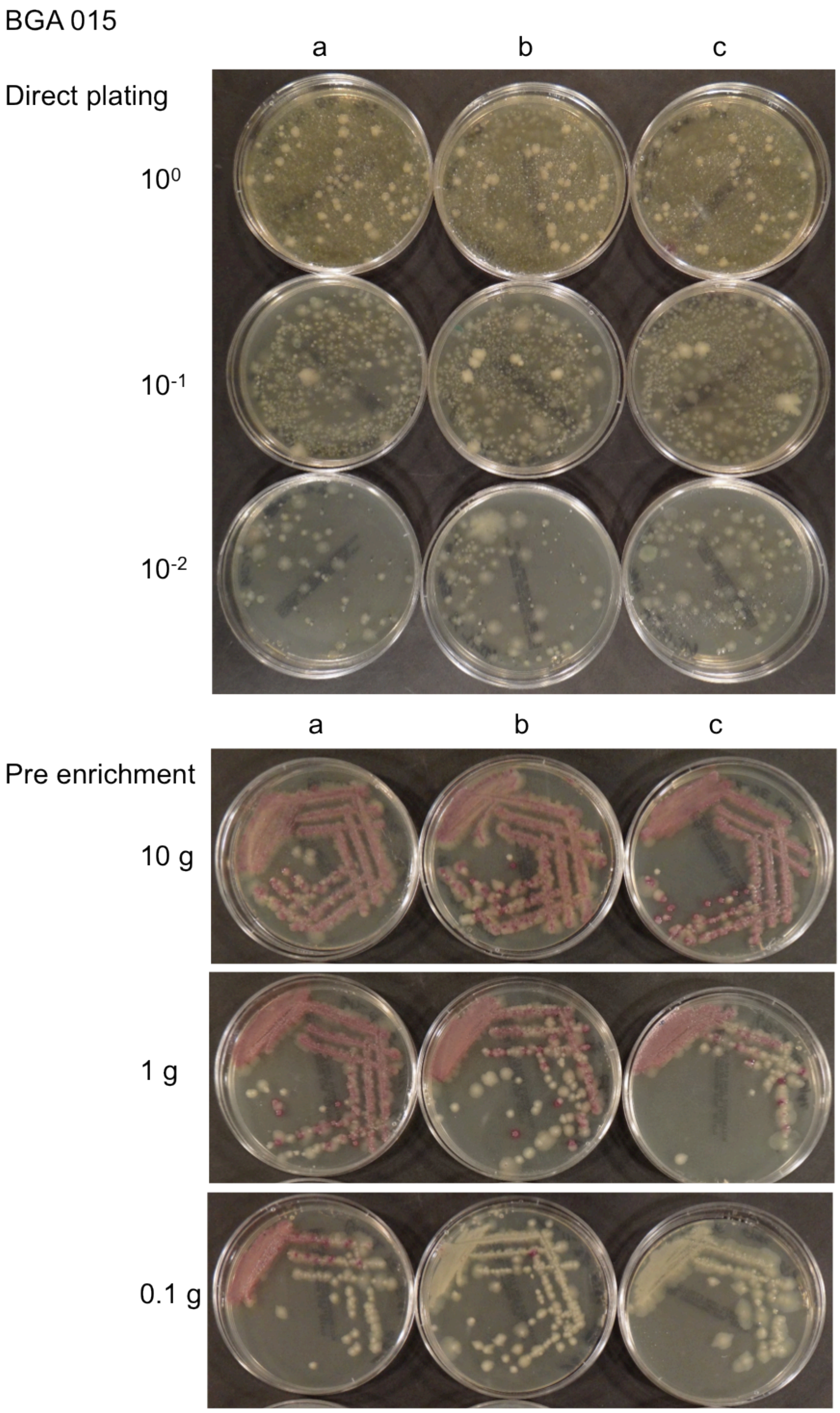

Figure S1. Overview of growth on CHROMagar ESBL after direct plating (sample dilutions 10^0 to 10^{-2}) and after pre-enrichment of 0.1, 1, and 10 g.
