# Supplementary material for: Improved Detection of Extended Spectrum Beta-Lactamase (ESBL)-Producing Escherichia coli in Input and Output Samples of German Biogas Plants by a Selective Pre-Enrichment Procedure
Source: PLoS One. 2015 Mar 23;10(3):e0119791. doi: 10.1371/journal.pone.0119791 (PMC4370489; doi:10.1371/journal.pone.0119791)
Supplement: S2 Fig — (PDF) [file pone.0119791.s002.pdf]

	2012										2013								2014		2013								2014	
	BGA 001		BGA 002		BGA 005		BGA 006		BGA 012		BGA 001 Feb.		BGA 001 April		BGA 001 July		BGA 001 Oct.		BGA 001 Feb.		BGA 015 Feb		BGA 015 April		BGA 015 Aug.		BGA 015 Oct.		BGA 015 Feb	
BOX Type	Input	Output	Input	Output	Input	Output	Input	Output	Input	Output	Input	Output	Input	Output	Input	Output	Input	Output	Input	Output	Input	Output	Input	Output	Input	Output	Input	Output	Input	Output
					no *E.coli*				no *E.coli*			not investigated							not investigated			not investigated							not investigated	
1a																					6									
1b															9						8				5					
1c																					1									
1d																										2	5			
1e																				2										
2a															3						2									
2b															2	1														
2f																	5													
2g																	1													
2h																				2										
3a											7		1										1		1	5				
3b													1															1		
3c																							1							
3d											2																			
3e																							2							
3f																	3													
3g																				1										
4a													1	7	1															
4b													1																	
4c													1																	
4d													14	6									1							
5a													1																	
5b	1													1									1							
5c																							12							
5g																							2							
6a														1																
12a							1	1																						
12b							2																							
12c							2																							
12e			2																											
20a	1																													
30c																	3													
30e																	1													
30f																											1			
30h																				5										
30i																				1										
30j																				1										
30k																				3										
32b																	2													
60a															1															
60b																				4										

Figure S2. Distribution of *E. coli* genotypes (defined by BOX-PCR pattern) in input and output samples of the investigated biogas plants. Grey boxes indicated the presences of a specific genotype. Numbers in Boxes represent the number of individual isolates which were analyzed.
