# Supplementary material for: Improved Detection of Extended Spectrum Beta-Lactamase (ESBL)-Producing Escherichia coli in Input and Output Samples of German Biogas Plants by a Selective Pre-Enrichment Procedure
Source: PLoS One. 2015 Mar 23;10(3):e0119791. doi: 10.1371/journal.pone.0119791 (PMC4370489; doi:10.1371/journal.pone.0119791)
Supplement: S3 Fig — (PDF) [file pone.0119791.s003.pdf]

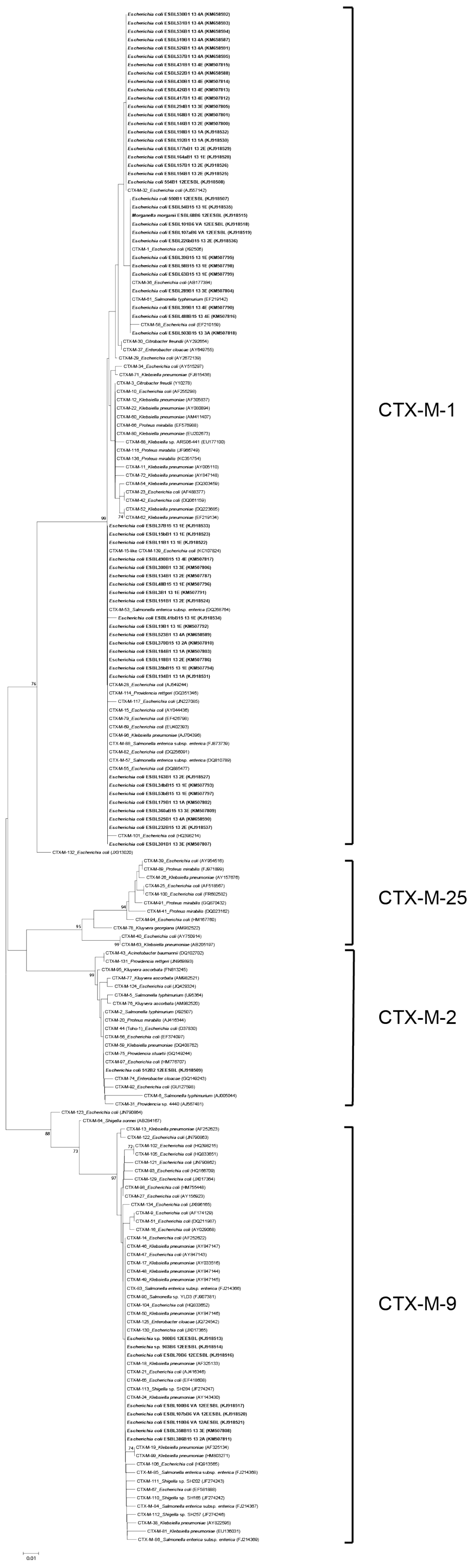
CTX-M-1
CTX-M-25
CTX-M-2
CTX-M-9
0.01

Figure S3 Phylogenetic assignment of CTX-M genes detected in ESBL isolates to main CTX-M groups based on partial amino acid sequence based phylogenetic analysis. A Neighbor-joining tree was calculated in MEGA5 based on the evolutionary JTT matrix and 100 re-samplings (bootstrap analysis). A total of 183 amino acid sequences were included in the analysis including a total of 4 different CTX-M-gene types. The phylogenetic analysis based on 165 amino acid sequence positions. Bar: 0.01 substitutions per amino acid sequence position.
