# Supplementary material for: Improved Detection of Extended Spectrum Beta-Lactamase (ESBL)-Producing Escherichia coli in Input and Output Samples of German Biogas Plants by a Selective Pre-Enrichment Procedure
Source: PLoS One. 2015 Mar 23;10(3):e0119791. doi: 10.1371/journal.pone.0119791 (PMC4370489; doi:10.1371/journal.pone.0119791)
Supplement: S4 Fig — (PDF) [file pone.0119791.s004.pdf]

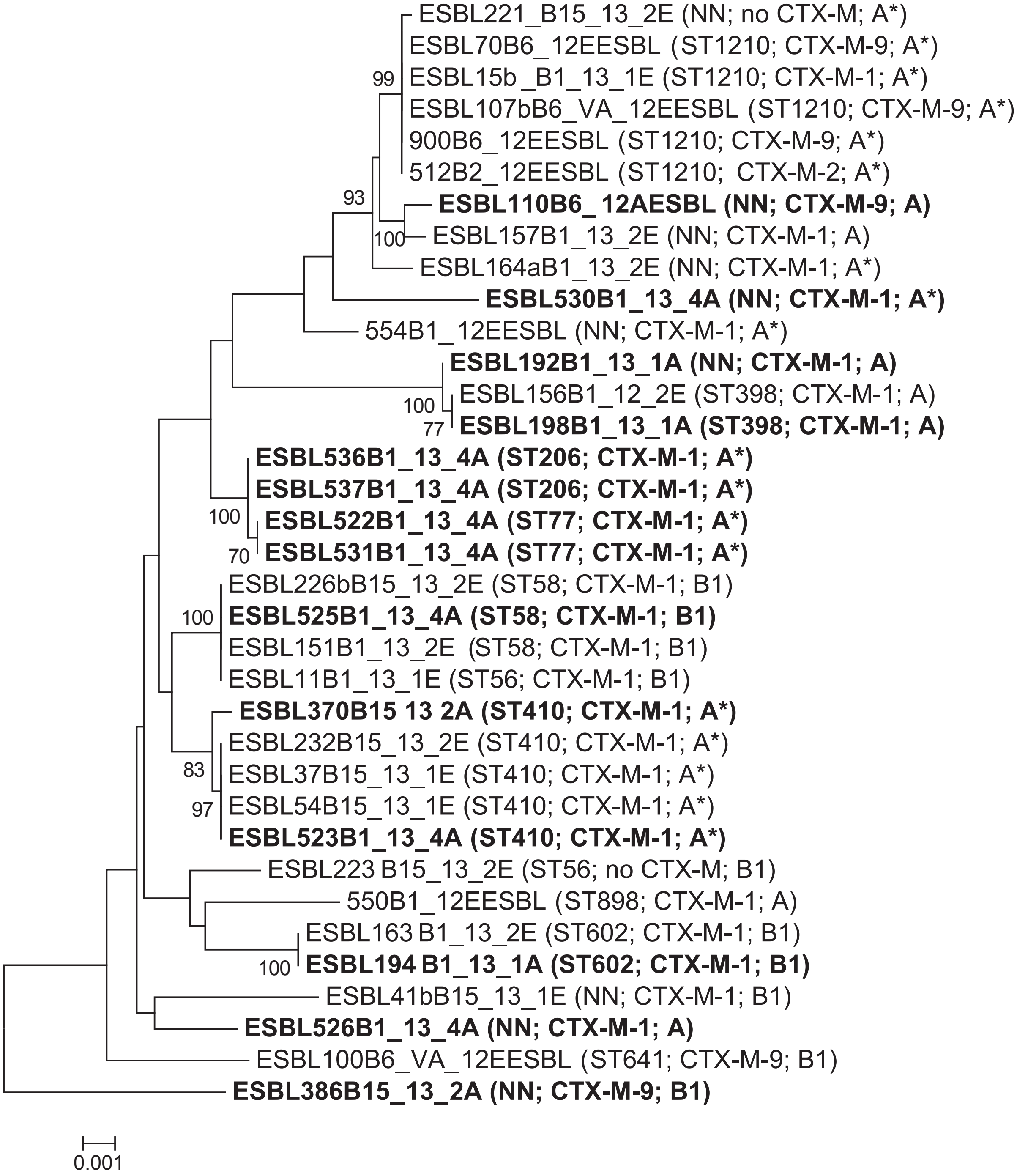

Figure S4. Multilocus sequence based maximum-likelihood tree based on concatenated nucleotide sequences showing the phylogenetic variety of *E. coli* isolates. All isolated from output samples are marked in bolt. Sequence types, CTX-M-groups and phylogenetic *E. coli* assignments are given in brackets. ST types were determined using the *E. coli* MLST database (http://mlst.warwick.ac.uk/mlst/dbs/Ecoli). Bar: 0.001 Nucleotide substitutions per sequence positions.
