# Supplementary material for: Improved Detection of Extended Spectrum Beta-Lactamase (ESBL)-Producing Escherichia coli in Input and Output Samples of German Biogas Plants by a Selective Pre-Enrichment Procedure
Source: PLoS One. 2015 Mar 23;10(3):e0119791. doi: 10.1371/journal.pone.0119791 (PMC4370489; doi:10.1371/journal.pone.0119791)
Supplement: S1 Table — (PDF) [file pone.0119791.s005.pdf]

Table S1 Overview of the composition of input samples and bioenergy crops from the different biogas plants investigated in 2012 (A) and 2013 (B)

A

**Biogas plant**	**Input sample composition (in percent)**	**Supplemented bioenergy crops (t per day)**
BGA 001	75% slurry (3:1 fatting pig to dairy cattle)<br>25% manure (6:0.5 cattle to laying henns)	7 corn<br>1.2 forage rye
BGA 002	50% slurry (3:1 fatting pig to dairy cattle)<br>50% manure (1:1:2 horse to chicken to cattle	8 corn<br>8 forage rye
BGA 005	73% dairy cattle slurry<br>27% manure (2:1 cattle to fatting chicken)	9 corn<br>0.6 grass silage
BGA 006	83% slurry (1:1 dairy cattle to breeding sow)<br>17% dairy cattle manure	9.3 corn<br>0.3 grass silage<br>6 rye silage<br>0.5 barley
BGA 012	100% chicken manure	25.5 corn

B

	**Time point**	**Input sample composition (in percent)**	**Supplemented bioenergy crops (t per day)**
BGA 001	February	43% slurry (cattle)<br>57% manure (5:1 cattle to chicken)	7.5 corn<br>1.5 forage rye
	April	49% slurry (3:1 pig to cattle)<br>51% manure (5:1 cattle to chicken)	6.5 corn<br>1.5 forage rye
	July	46% slurry (pig)<br>54% manure (6:1 cattle to chicken)	6 corn<br>2 forage rye
	October	54% slurry (20:1 cattle to pig)<br>55% manure (6:1 cattle to chicken)	7 corn<br>2 grass sillage
BGA 015	February	70% slurry (cattle)<br>30% manure (2.5:1 chicken to horse)	6.5 corn<br>1 grass silage
	April	60% slurry (cattle)<br>40% manure (3:1 chicken to horse)	5.5 corn<br>0.5 grass silage
	August	76% slurry (cattle)<br>24% manure (4:1 chicken to horse)	2 corn<br>6 rye silage<br>1 crushed corn
	October	71% slurry (cattle)<br>29% manure (4:1 chicken to horse)	6 corn<br>3 grass sillage<br>1 crushed corn
